# Exploratory effects of coffee-derived exosome-like nanoparticles in a fibrillar amyloid-beta 1–42-induced neurodegenerative rat model: evidence from fluorodeoxyglucose positron emission tomography and phosphorylated tau/amyloid precursor protein expression

**DOI:** 10.7717/peerj.21739

**Published:** 2026-09-29

**Authors:** Yusuf Kucukbagriacik, Gulistan Sanem Saribas, Kahraman Ates, Cenan Ozturk, Eren Kahyaoglu, Sezen Kislal, C. Ceren Akgul, A. Ozgur Karacalioglu, Cuneyt Goksoy, Murat Kantarcioglu

**Affiliations:** 1 Department of Biophysics, Gulhane Medical Faculty, University of Health Sciences, Ankara, Turkey; 2 Department of Histology and Embryology, Gulhane Medical Faculty, University of Health Sciences, Ankara, Turkey; 3 Department of Biology, Faculty of Science, Ankara University, Ankara, Turkey; 4 Department of Molecular Biology and Genetics, Faculty of Arts and Sciences, Nevsehir Hacı Bektaş Veli University, Nevsehir, Turkey; 5 Department of Psychology, Faculty of Arts and Science, Middle East Technical University, Ankara, Turkey; 6 Department of Nuclear Medicine, Gulhane Medical Faculty, University of Health Sciences, Ankara, Turkey; 7 Department of Gastroenterology, Faculty of Medicine, Yuksek Ihtisas University, Ankara, Turkey

**Keywords:** Alzheimer’s disease, Amyloid-beta, CELNs, Coffee-derived exosome-like nanoparticles, FDG-PET

## Abstract

**Background:**

Alzheimer’s disease (AD) is a progressive neurodegenerative disorder characterized by amyloid-beta 1–42 (Aβ_1–42_) accumulation, tau pathology, and metabolic dysfunction. This exploratory study evaluated the effects of coffee-derived exosome-like nanoparticles (CELNs) on selected metabolic and histopathological endpoints in a fibrillar Aβ_1–42_-induced neurodegenerative rat model.

**Methods:**

Fifty male *Sprague–Dawley* rats were randomly divided into five groups (*N* = 10 each): Control (C), sham (SF), a fibrillar Aβ_1–42_-induced neurodegenerative rat model (AD), AD treated with a low dose of CELNs (AD + LE, 10 mg/kg/day), and AD treated with a high dose of CELNs (AD + HE, 20 mg/kg/day) for 14 days. CELNs were quantified and characterized using the bicinchoninic acid method, scanning electron microscopy, and nanoparticle tracking analysis (NTA). Micro-positron emission tomography/computed tomography fluorodeoxyglucose (MicroPET/CT FDG) imaging of the central nervous system was performed in all groups at the end of treatment. Subsequently, all animals were sacrificed, and hippocampal tissues were subjected to histopathological analysis.

**Results:**

MicroPET imaging demonstrated a significant, dose-dependent increase in cerebral glucose metabolism in AD rats treated with CELNs compared with the untreated AD group. Histopathological evaluations were consistent with the imaging data: in AD groups receiving CELNs, phospho-tau and beta-amyloid precursor protein expression were significantly reduced in the hippocampal cornu ammonis 2 (CA2) and dentate gyrus regions compared with the untreated AD group.

**Conclusions:**

In this exploratory fibrillar Aβ_1–42_-induced neurodegenerative rat model, oral CELN administration was associated with dose-dependent partial improvements in cerebral glucose metabolism and region-specific reductions in hippocampal phosphorylated tau (p-Tau) and amyloid precursor protein (APP) immunoreactivity. However, these are preliminary biomarker-level findings that support further investigation of CELNs; definitive confirmation of functional and cognitive benefits requires additional behavioral, biodistribution, and experimental validation.

## Introduction

The development of Alzheimer’s disease (AD) involves complex molecular mechanisms influenced by biochemical and environmental factors. This process leads to neurodegeneration and cognitive decline by contributing to the accumulation of amyloid-beta plaques, disruption of the blood-brain barrier, increased oxidative stress, tau protein hyperphosphorylation, and mitochondrial dysfunction (Afsar et al., 2023). With the aging global population, the prevalence of Alzheimer’s disease continues to rise, making it a major public health concern.

Exosomes are nano-sized vesicles (50–150 nm) secreted by various living cells, possessing diverse immunological, interactive, and regulatory functions (Powis et al., 2011). Enclosed by a lipid bilayer, these vesicles carry small non-coding RNAs such as microRNAs and tRNA fragments with potential regulatory activity. They also encapsulate proteins and lipids characteristic of their parent cells (Vojtech et al., 2014). In recent years, extracellular vesicles, including exosomes, have received considerable attention as mediators of intercellular communication and contributors to the pathogenesis of neurodegenerative diseases. By transferring molecular information, they modulate the functions of recipient cells.

Plant-derived exosome-like nanoparticles (PELNs) have emerged as promising therapeutic agents because of their biocompatibility, low toxicity, and ability to cross biological barriers (Maitra, Sarkar & Dhara, 2024). These nano-scale vesicles (30–150 nm) contain biomolecules that can influence plant growth and defense mechanisms (Cho et al., 2022). PELNs have demonstrated potential applications in the treatment of inflammatory diseases (Wei et al., 2023), cancer, and microbial infections (Maitra, Sarkar & Dhara, 2024). Furthermore, they can be engineered for applications in targeted drug delivery, gene therapy, and tissue regeneration (Dutta et al., 2025).

Coffee, one of the most widely consumed beverages globally, contains numerous bioactive compounds, minerals, and vitamins (Samoggia & Riedel, 2019). Regular coffee consumption has been associated with a reduced risk of cardiovascular disease, type 2 diabetes, liver disorders, depression, and neurodegenerative conditions (Poole et al., 2017). A prospective study indicated that protective lifestyle factors, including coffee consumption and physical activity, may reduce the risk of Alzheimer’s disease (Lindsay et al., 2002). A pooled estimate from this study suggested that coffee consumption lowers Alzheimer’s disease risk, underscoring the need for further prospective research (Quintana et al., 2007). Optimal intake has been reported to provide protective benefits against Alzheimer’s disease, with important implications for disease prevention (Hussain et al., 2018). Notably, coffee-derived exosome-like nanoparticles (CELNs) were first identified in 2022, and their properties have since been characterized (Kantarcıoğlu et al., 2023).

Coffee-derived exosome-like nanoparticles are operationally defined in this study as plant-derived extracellular vesicle-like particles isolated from *Coffea arabica* and characterized by nanoscale size distribution, vesicle-like morphology, and measurable protein content. Because plant-derived vesicles do not necessarily share the canonical biogenesis pathway or mammalian exosomal markers such as CD9, CD63, and CD81, the term “exosome-like nanoparticles” is used descriptively rather than as evidence of mammalian-type exosome identity. The potential relevance of CELNs in neurodegenerative models is based on their nanoscale structure, plant-derived molecular cargo, oral administration feasibility, and reported biological activity of plant-derived vesicle-like particles in inflammatory and neural injury models. However, direct cellular uptake, brain biodistribution, and cargo-mediated mechanisms were not examined in the present study and are therefore discussed as future research directions.

The present study aimed to explore the potential of CELNs in an experimentally a fibrillar amyloid-beta 1–42 (Aβ_1–42_)-induced neurodegenerative rat model as a preliminary, hypothesis-generating investigation. A dietary supplement containing CELNs was administered orally *via* gavage at two doses (10 and 20 mg/kg/day) for 14 consecutive days. To the best of our knowledge, this is the first study to investigate the effects of CELNs in a fibrillar Aβ_1–42_-induced neurodegenerative rat model. We assessed their impact on neuropathological markers through immunohistochemical analysis and on cerebral glucose metabolism using fluorodeoxyglucose positron emission tomography (FDG-PET) imaging. These exploratory findings are intended to provide a foundation for future research employing plant-derived nanovesicles for neurodegenerative diseases and should be interpreted with caution, as behavioral, biodistribution, or direct cellular uptake analyses were not included in this study.

## Materials and Methods

### Reagents and chemicals

Amyloid-beta (Aβ_1–42_) peptide (Cat. No. A15420; Adooq Bioscience, Irvine, CA, USA) was used to induce a fibrillar Aβ_1–42_-induced neurodegenerative rat model. Ketamine (Keta-Control, Arion İlaç, Türkiye) and xylazine (Xylazinbio 2%; Bioveta, Ivanovice na Hané, Czech Republic) were administered for anesthesia. Saline solution (0.9% isotonic NaCl; Polifarma, Polifleks, Türkiye) was used for peptide dilution and control injections. CELNs (AYE Exocure, Ankara, Türkiye) were obtained for evaluation in this model. Fluorodeoxyglucose (FDG; PETNET Solutions, Siemens Healthineers, Malvern, PA, USA) was used for metabolic brain imaging with micro-positron emission tomography/computed tomography (MicroPET/CT).

The bicinchoninic acid (BCA) protein assay kit (Cat. No. P011; Atlas Biyoteknoloji, Ankara, Türkiye) was used for protein quantification. Primary antibodies against phospho-tau (p-Tau; Cat. No. AF3148; Affinity Biosciences, Cincinnati, OH, USA) and beta-amyloid precursor protein (APP; Cat. No. AF6084; Affinity Biosciences, Cincinnati, OH, USA) were used for immunohistochemistry. Biotinylated secondary antibody (Cat. No. TP-125-BN; Thermo Scientific, Waltham, MA, USA), streptavidin–peroxidase complex (Cat. No. TS-125-HR; Thermo Scientific, Waltham, MA, USA), diaminobenzidine (DAB; Cat. No. DABC-004; Spring Bioscience, Pleasanton, CA, USA), and amino ethyl carbazole (AEC; Cat. No. PL-125-HA; PatoLab, İstanbul, Turkey) were employed for signal detection. All reagents were of analytical grade and purchased from commercial suppliers.

### Animal procurement, housing and experimental workflow

All experimental procedures were approved by the KOBAY DHL A.Ş. Local Ethics Committee (KOBAY Experimental Animals Laboratory Industry and Trade Inc., Ankara, Türkiye), Ankara, Türkiye (Protocol No. 685; Approval date: 9 November 2023), and were conducted in accordance with national regulations and the ARRIVE 2.0 guidelines.

Adult male Sprague–Dawley rats were obtained from KOBAY Experimental Animals Laboratory (Ankara, Türkiye). Stereotaxic surgical procedures for model induction were performed by the authors at the laboratories of the University of Health Sciences. Following surgery, animals were transferred back to KOBAY and housed under controlled conditions during the 14-day treatment period. Rats were housed in plexiglass cages under controlled conditions (temperature: 22 °C; humidity: 50 ± 5%; 12-h light/dark cycle) with *ad libitum* access to food and water. Humane endpoints were defined as >20% body weight loss or severe deterioration in general condition; no animals met these criteria. Anesthesia was induced with intraperitoneal ketamine (90 mg/kg) and xylazine (10 mg/kg). At study termination, animals were euthanized with an overdose of anesthetics followed by decapitation under deep anesthesia to minimize pain and distress.

Throughout the treatment phase, all interventions were administered once daily at the same time, by the same researcher, and in the same experimental setting. Animals were housed in group-specific cages, with each experimental group kept separately, while all cages were located within the same room to minimise environmental variability.

MicroPET/CT imaging analyses were conducted at the laboratories of Ankara University, after which animals were returned to KOBAY for euthanasia in accordance with the approved ethical protocol. Tissue dissection and separation were subsequently performed by the authors, and histopathological and immunohistochemical analyses were carried out at the laboratories of the University of Health Sciences.

### Experimental design

The study included 50 adult male *Sprague–Dawley* rats (8–12 weeks, 250–350 g). Animals were randomly assigned to five groups (*N* = 10 each; Table 1). Randomization ensured baseline comparability and minimized allocation bias.

**Table 1:** Experimental groups.

Group name	Sample size (*n*)	Alzheimer’s disease model	CELNs treatment
Control group (C)	10	–	–
Saline solution group (SF)	10	SF	–
Alzheimer’s disease model control group (AD)	10	Aβ_1–42_	–
Alzheimer + low-dose CELNs treatment group (AD + LE)	10	Aβ_1–42_	10 mg/kg/day for 14 days
Alzheimer + high-dose CELNs treatment group (AD + HE)	10	Aβ_1–42_	20 mg/kg/day for 14 days

CELNs were supplied in powder form and freshly prepared daily by dissolving in distilled water at concentrations of 10 and 20 mg/mL. For treatment, one mL of either solution was administered by oral gavage using a 16-gauge curved stainless-steel gavage needle (5-cm length) once daily at a fixed time (12:00 p.m.) for 14 consecutive days. All administrations were performed by the same trained researchers to ensure consistency. Rats were not fasted before administration and were gently hand-restrained to minimize stress.

### Experimental timeline

Aβ_1–42_ aliquots were incubated at 37 °C for 7 days before stereotaxic injection. Day 0: Bilateral intracerebroventricular injection of saline or Aβ_1–42_ was performed. Day 1 to Day 14: CELNs were administered once daily by oral gavage at 10 or 20 mg/kg/day in the AD + LE and AD + HE groups, respectively. Control, SF, and AD groups received no CELN treatment. Day 14: FDG-PET/CT imaging was performed after the final treatment period. Immediately after imaging: Animals were euthanized under deep anesthesia, and brain tissues were collected for histological and immunohistochemical analyses.

Control group (C): No intervention was applied. No CELN treatment was administered.

Saline solution group (SF): The animals were administered physiological saline solution (5.0 µL) bilaterally into the lateral ventricles *via* stereotaxic surgery, which served as the vehicle control for amyloid-beta delivery. No CELN treatment was administered.

Fibrillar Aβ_1–42_-induced neurodegenerative rat model (AD): The animals were administered amyloid-beta (5.0 µL) bilaterally into the lateral ventricles *via* stereotaxic surgery. No CELN treatment was administered.

Fibrillar Aβ_1–42_-induced neurodegenerative rat model + low-dose CELN treatment group (AD + LE): The animals were administered amyloid-beta (5.0 µL) bilaterally into the lateral ventricles *via* stereotaxic surgery. Additionally, CELNs were given to the animals at a dose of 10 mg/kg/day for 14 consecutive days.

Fibrillar Aβ_1–42_-induced neurodegenerative rat model + high-dose CELN treatment group (AD + HE): The animals were administered amyloid-beta (5.0 µL) bilaterally into the lateral ventricles *via* stereotaxic surgery. Additionally, CELNs were given to the animals at a dose of 20 mg/kg/day for 14 consecutive days.

### Surgery and induction of the fibrillar Aβ_1–42_-induced neurodegenerative rat model

All surgical procedures were performed at the Department of Biophysics, Gulhane Medical Faculty. Bilateral intracerebroventricular injection of Aβ_1–42_ was used to induce AD in the relevant groups.

Aβ_1–42_ (AdooQ Bioscience, Irvine, CA, USA) was dissolved in 1,000-μL physiological saline, aliquoted into 40-μL stocks, and stored at −20 °C until use. Before injection, aliquots were thawed in a 37 °C incubator for 7 days. Based on the incubation conditions used in this study, the injected Aβ_1–42_ preparation is best interpreted as a fibril-enriched Aβ_1–42_ preparation rather than a soluble oligomeric preparation. The injected material was not separately characterized by electron microscopy, DLS, or biochemical aggregation assays; therefore, the model is referred to throughout the revised manuscript as a fibrillar Aβ_1__–42_-induced neurodegenerative rat model.

Following intraperitoneal anesthesia with ketamine (90 mg/kg) and xylazine (10 mg/kg), the rats’ heads were shaved and fixed in a stereotaxic frame. A midline scalp incision was made, the periosteum reflected, and 0.8-mm burr holes drilled. The stereotaxic coordinates for the lateral ventricles were 0.8 mm posterior to bregma, 1.4 mm lateral to the sagittal suture, and 4 mm below the dura mater. Aβ_1–42_ (5.0 μL) was drawn into a Hamilton syringe and injected bilaterally over 5 min. To prevent backflow, the syringe was left in place for 2 min before withdrawal. The incision was sutured, and antibiotic cream was applied to the wound to prevent infection (Aru et al., 2023).

### CELNs characterization

This study characterized a dietary supplement containing exosome-like nanoparticles derived from *Coffea arabica* (AYE Exocure, Ankara, Türkiye) using SEM, nanoparticle tracking analysis (NTA), and BCA analyses.

The CELN preparation used in this study was obtained from a single production batch (Batch/Lot No: Batch02022024CE). Because all animals received material from the same batch, potential batch-to-batch variation was minimized within this experiment. The preparation was then administered orally at 10 or 20 mg/kg/day in the fibrillar Aβ_1–42_-induced model to assess treatment-associated biomarker-level effects. The CELN preparation was produced by AYE Exocure using a standardized, proprietary manufacturing process developed for *Coffea arabica*-derived vesicle-like nanoparticle preparations. The production workflow included controlled processing of *Coffea arabica*-derived material, removal of coarse plant residues, enrichment of the nanoscale particle-containing fraction, and preparation of the final CELN-containing material under batch-controlled conditions. The final batch used in the present study was characterized by SEM, NTA, and BCA before animal administration. No additional production batch was used in the *in vivo* experiment.

### Characterization of CELNs by scanning electron microscopy

Scanning electron microscopy (SEM) was used to evaluate the morphological characteristics and membrane integrity of CELNs. Analyses were conducted in two independent laboratories to improve accuracy and validate results: Hacettepe University Advanced Technologies Application and Research Center (TESCAN GAIA3 SEM; HUNITEK, Ankara, Turkey) and Ankara University Institute of Nuclear Sciences (Ankara, Turkey; ZEISS EVO40 SEM). Imaging was performed at magnifications of 25K×, 30K×, 50K×, and 80K× in both laboratories. Before analysis, CELNs were coated with gold–tungsten nanoparticles to enhance electron conductivity. SEM images confirmed that PELNs exhibited spherical morphology and preserved membrane integrity. CELN size was measured using ImageJ software, and the average values were consistent with those reported in the literature.

### CELNs quantitation (NTA and BCA)

Nanoparticle tracking analysis was employed to determine the size distribution and concentration of CELNs. NTA is a highly sensitive technique that measures hydrodynamic diameters and numerical concentrations of particles by tracking Brownian motion. Analyses were performed at HUNITEK using the Nanosight NS300 (Malvern Panalytical, Worcestershire, United Kingdom).

To assess total protein concentration, the BCA assay was conducted using the ABP Bioscience BCA Protein Assay Kit (P011). This widely used colorimetric method quantifies protein through the reduction of Cu^2+^ to Cu^1+^ in an alkaline environment, followed by detection with bicinchoninic acid. CELN samples were prepared at a concentration of 100 mg/mL in distilled water. Protein concentration was then measured according to the manufacturer’s protocol.

### FDG-PET imaging and study design

FDG-PET imaging, following the methodology of Mansur et al. (2021), and previously validated protocols demonstrating low test–retest variability, was employed to evaluate brain glucose metabolism in animals subjected to the AD model, treatment, and control conditions (Lu et al., 2016).

PET image quantification was conducted using standardized protocols for region-of-interest selection and signal intensity measurement. Analyses were performed under blinded conditions to minimize bias. Before imaging, rats were fasted for 12 h with free access to water. To reduce movement, anesthesia was induced with 0.5 cc of Xylazine:Ketamine (1:9). Each rat received an intravenous injection of 0.1 cc 1.2 mCi 18F-FDG ([^18^F] FDG-PET) *via* the tail vein. Following injection, the animals were kept in a dark, quiet environment for 30 min to allow optimal FDG distribution in brain tissue while minimizing stress and movement.

Scanning was performed at the Department of Nuclear Medicine, Ankara University, using the Sedecal Super Argus MicroPET/CT scanner. Images were analyzed with the IDL Virtual Machine Imaging Software. Glucose metabolism across brain regions was compared by calculating SUVmax and SUVmean (standardized uptake values).

### Histochemical processing and methodology

Brain tissue samples were fixed in 4% paraformaldehyde for 72 h for light microscopic examination. Following routine tissue processing, samples were embedded in paraffin blocks. Sections of approximately 4–5 μm thickness were obtained and stained with hematoxylin and eosin (H&E) for general morphological evaluation. Stained sections were examined and photographed using a light microscope.

Histological analysis with H&E staining focused on parasagittal brain sections from each group, particularly the cerebral cortex and hippocampal regions (CA1, CA2, CA3, and dentate gyrus). Structural and pathological alterations related to model induction and CELN administration were assessed qualitatively and compared with control groups.

### Immunohistochemical method

For immunohistochemistry, 4-µm sections were obtained from paraffin blocks and mounted on poly-L-lysine-coated slides. Sections were incubated at 61 °C for 20 min to facilitate deparaffinization, followed by immersion in xylene twice for 20 min each. They were then passed through a graded alcohol series and washed with distilled water. Antigen retrieval was performed in citrate buffer (pH 6.0), and the sections were cooled for 20 min at room temperature. After three washes with PBS (pH 7.4), endogenous peroxidase activity was blocked with 3% hydrogen peroxide for 20 min. Nonspecific binding was reduced with UltraV block for 5 min.

Slides were incubated overnight at +4 °C with primary antibodies: Phospho (p)-Tau (AF3148, rabbit polyclonal; Affinity Biosciences, Cincinnati, OH, USA) and beta-APP (AF6084, rabbit polyclonal; Affinity Biosciences, Cincinnati, OH, USA), both at 1:200 dilution. After primary incubation, a biotinylated secondary antibody (Cat: TP-125-BN, Lab Vision; Thermo Scientific, Waltham, MA, USA) was applied. Following PBS washes, sections were incubated for 10 min with streptavidin–peroxidase enzyme complex (Cat: TS-125-HR, Lab Vision; Thermo Scientific, Waltham, MA, USA). Chromogenic detection used diaminobenzidine (DAB; Cat: DABC-004; Spring Bioscience, Pleasanton, CA, USA) for p-Tau and aminoethyl carbazole (AEC; PL-125-HA; PatoLab, İstanbul, Turkey) for APP. Mayer’s hematoxylin served as the counterstain. Digital images of immunohistochemically stained sections were acquired using a computer-assisted light microscopy imaging system under identical illumination, magnification, and exposure settings for all samples. Quantitative analysis of p-Tau and APP immunoreactivity was performed using ImageJ software (National Institutes of Health, Bethesda, MD, USA). AEC- and DAB-positive immunostaining were quantified as the percentage of positive staining area (% positive area) within predefined regions of interest (ROIs). Images were processed using the Color Threshold function in ImageJ to selectively identify AEC red and DAB brown chromogen positivity. Threshold parameters were initially optimized using negative control sections to minimize nonspecific background staining and were subsequently kept constant for all analyzed images. Background artifacts and non-specific staining were excluded from the analysis. Quantitative data were expressed as the mean percentage of positive stained area for each group. All image analyses were performed by an investigator blinded to the experimental groups.

### Statistical analysis

*A priori* power analysis (G*Power 3.1) for a one-way fixed-effects analysis of variance (ANOVA) with five groups (equal allocation) indicated that *N* = 80 was required to detect a large effect (Cohen’s f = 0.40) with α = 0.05 and 80% power. Considering feasibility and ethical constraints, the study proceeded with *N* = 50 (*N* = 10 per group). A sensitivity analysis for *N* = 50 (α = 0.05; five groups) showed 80% power to detect an omnibus effect of approximately f ≈ 0.52, indicating sensitivity to very large effects only.

Normality of continuous variables was assessed graphically and with the Shapiro–Wilk test. Descriptive statistics were presented as mean ± standard deviation, median, and minimum and maximum values. Histological and microPET/CT data were analyzed using one-way ANOVA for variables meeting parametric assumptions. When significant differences were found, Bonferroni *post hoc* tests identified the source. For nonparametric data, the Kruskal–Wallis test was applied, with significant findings followed by Bonferroni-adjusted Mann–Whitney pairwise comparisons.

All statistical analyses were performed with IBM SPSS Statistics version 21 (IBM Corp., New York, NY, USA). A *p* value ≤ 0.05 was considered statistically significant.

## Results

### Characterization of CELNs

Quantitative and qualitative analyses are essential for understanding the biological functions and therapeutic potential of CELNs. In this context, NTA and SEM provide reliable methods for characterizing exosomes, yielding reproducible data on their size and concentration (Mehdiani et al., 2015; Sokolova et al., 2011). NTA offers a rapid and efficient approach for monitoring exosomes in diverse biological samples (Soo et al., 2012). However, these techniques do not define molecular cargo composition or plant-specific exosome-like markers.

Scanning electron microscopy imaging showed that CELNs exhibited spherical and cup-shaped morphologies consistent with typical PELNs. The obtained SEM images (Figs. 1A, 1B) confirmed their structural integrity and purity. These findings demonstrate the suitability of CELNs for characterization and subsequent analysis.

**Figure 1:**
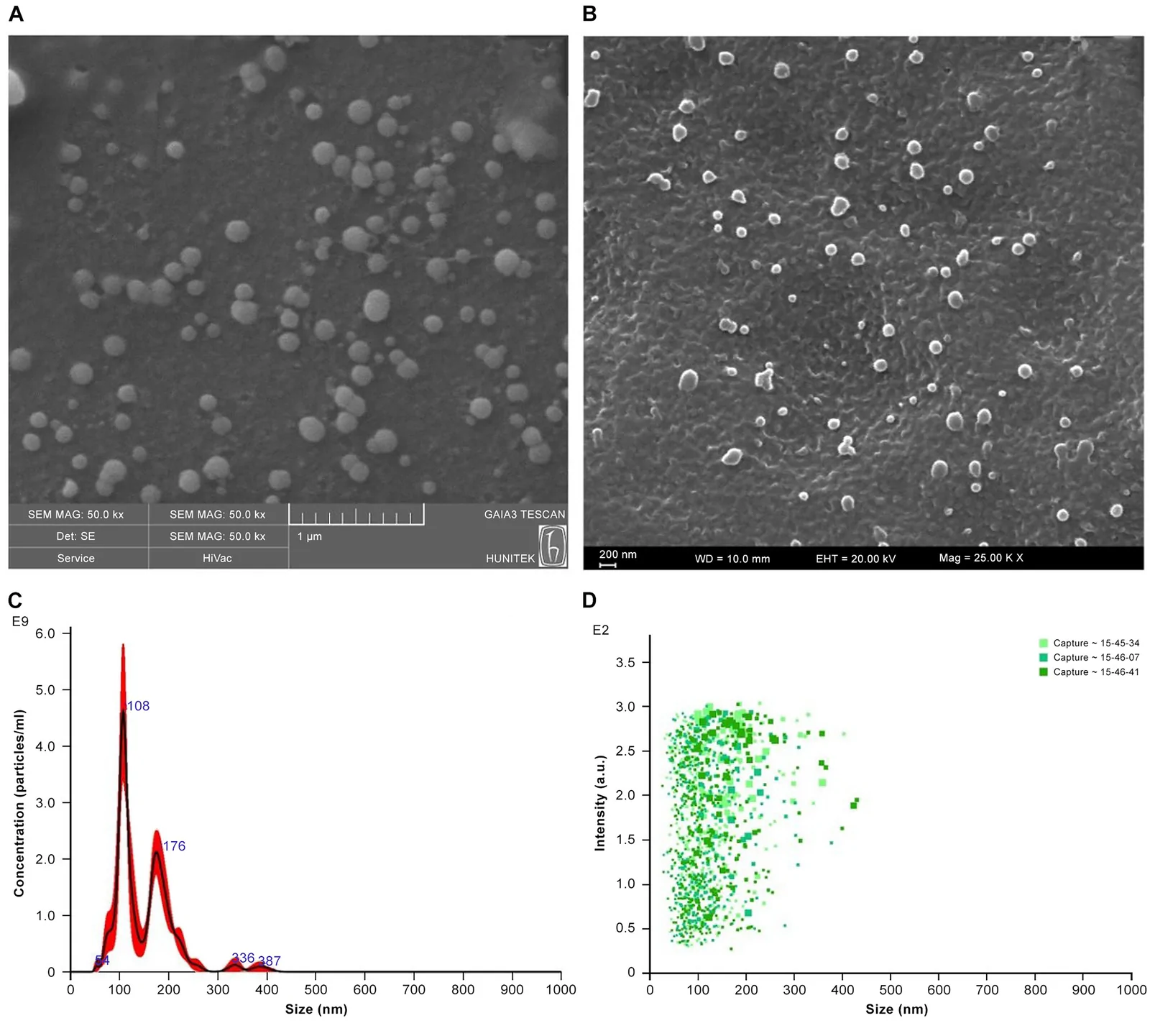
Morphological, size, and purity analysis of CELNs. (A, B) SEM images demonstrate homogeneous distribution and spherical morphology. (A) Image at 50.0k× magnification (Hacettepe University). (B) Image at 20.0k× magnification (Ankara University). Both confirm size distribution predominantly within 100–150 nm. (C, D) NTA results showing size distribution and concentration. (C) Particle size distribution indicates that CELNs are most abundant within the 100- to 190-nm range. (D) A scatter plot of particle intensity *vs*. size confirms a homogeneous distribution.

Purity was assessed by calculating the ratio of particle number (determined by NTA) to protein concentration (measured by the BCA assay). The mean CELN diameter was 149.2 ± 6.1 nm, with the most frequently observed size being 109.4 ± 3.7 nm. The total particle concentration was 2.50 × 10^11^ particles/mL (Figs. 1C, 1D). These data indicate nanoscale particle distribution consistent with coffee-derived exosome-like nanoparticle preparations.

The CELN sample, initially prepared at 100 mg/mL in distilled water, showed a total protein concentration of 85.91 mg/mL. This result indicates a protein-containing nanoparticle preparation. However, the present characterization remains partial because plant-specific markers such as TET8/PEN1, caffeine and chlorogenic acid quantification, proteomic profiling, lipidomic profiling, and RNA cargo analyses were not performed.

### FDG-PET imaging results

As shown in Figs. 2A, 2B, no statistically significant differences were observed between the C and SF groups, indicating that physiological saline did not affect the measured parameters. In contrast, the AD group exhibited significant decreases compared with both C and SF groups, supporting successful induction of metabolic impairment in a fibrillar Aβ_1–42_-induced neurodegenerative rat model.

**Figure 2:**
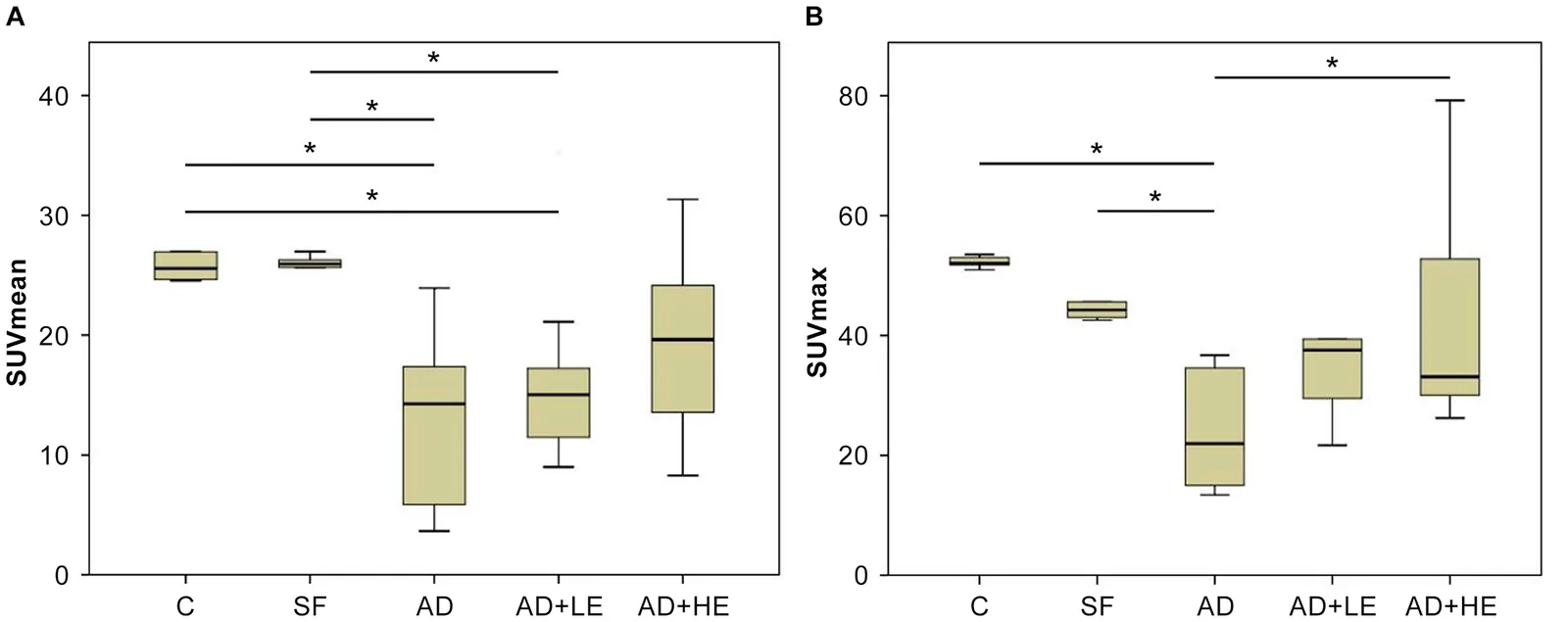
Analysis of SUV values across experimental groups. (A) SUVmean values. (B) SUVmax values. *p < 0.05 indicates a statistically significant difference between the compared groups.

In Fig. 2A, SUVmean values were significantly reduced in both AD and AD + LE groups compared with C and SF groups (*p* = 0.031). This reduction suggests that SUVmean values in the AD + LE group remained below control levels. The absence of significant differences between AD + HE, C, and SF groups indicates that SUVmean values in the AD + HE group were closer to control-range values. However, because AD + HE did not differ significantly from the untreated AD group for SUVmean, this parameter should be interpreted cautiously and does not establish a definitive dose-related effect.

As shown in Fig. 2B, SUVmax values were significantly reduced in the AD + LE group compared with the C and SF groups (*p* = 0.024), similar to the AD group. By contrast, the AD + HE group showed significantly higher SUV values than the AD group (*p* = 0.018), approaching levels observed in the C and SF groups. These results indicate treatment-associated differences in selected FDG-PET parameters, particularly for SUVmax.

MicroPET/CT analyses further supported these findings. Significant differences were observed in both SUVmean and SUVmax values among groups. Comparisons showed that the AD + HE group differed significantly from the AD group and was more effective than the AD + LE group in approximating values of the SF group. These findings indicate partial treatment-associated metabolic differences but do not establish behavioral improvement or disease-modifying activity.

The microPET/CT images (Fig. 3) demonstrate metabolic brain activity across experimental groups. Warmer colors (yellow, orange, and red) represent higher metabolic activity, while cooler, darker regions indicate reduced activity. The AD group exhibited markedly reduced brightness compared with all other groups, consistent with a decline in brain activity. In contrast, the AD + LE and AD + HE treatment groups showed increased brightness. These representative images should be interpreted together with the quantitative SUVmean and SUVmax analyses rather than as independent quantitative evidence.

**Figure 3:**
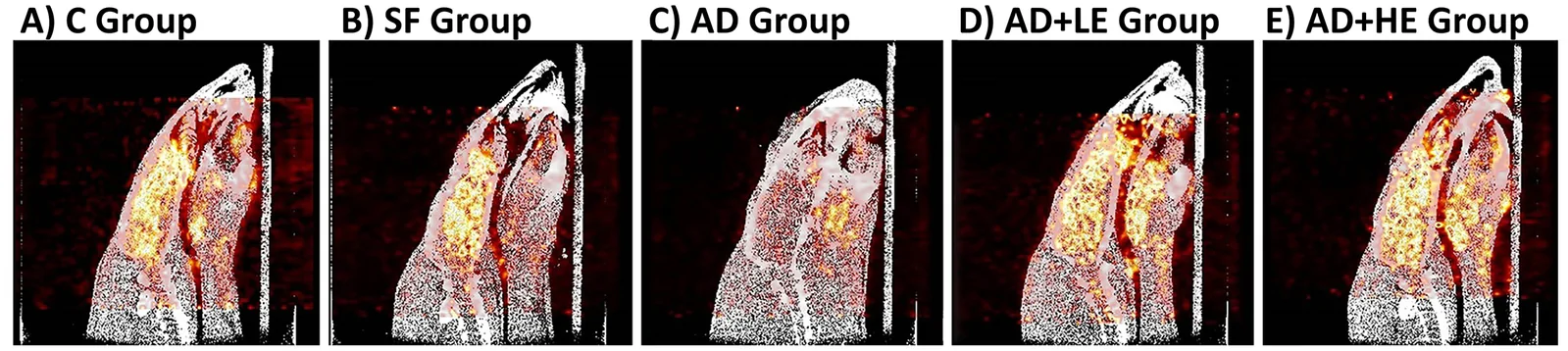
MicroPET/CT imaging of brain activity across groups. (A) C group, (B) SF group, (C) AD group, (D) AD + LE group, (E) AD + HE group. Warmer colors (yellow, orange, and red) represent higher metabolic activity; cooler colors (blue and dark) represent reduced activity. Images highlight distinct patterns of brain metabolism across groups, reflecting the effects of disease and treatment.

### Histomorphological findings

In parasagittal brain sections from the C and SF groups, the hippocampal formation—appearing as a curved structure on the medial surface of the temporal lobe—showed a normal histological appearance (Figs. 4A and 5A). The arrangement and morphology of hippocampal neurons and glial cells were preserved (Figs. 4A–4E and 5A–5E). In the cerebral cortex, neurons exhibited large, round vesicular nuclei, and both nuclei and cytoplasm of neurons and glial cells appeared normal (Figs. 4F and 5F).

**Figure 4:**
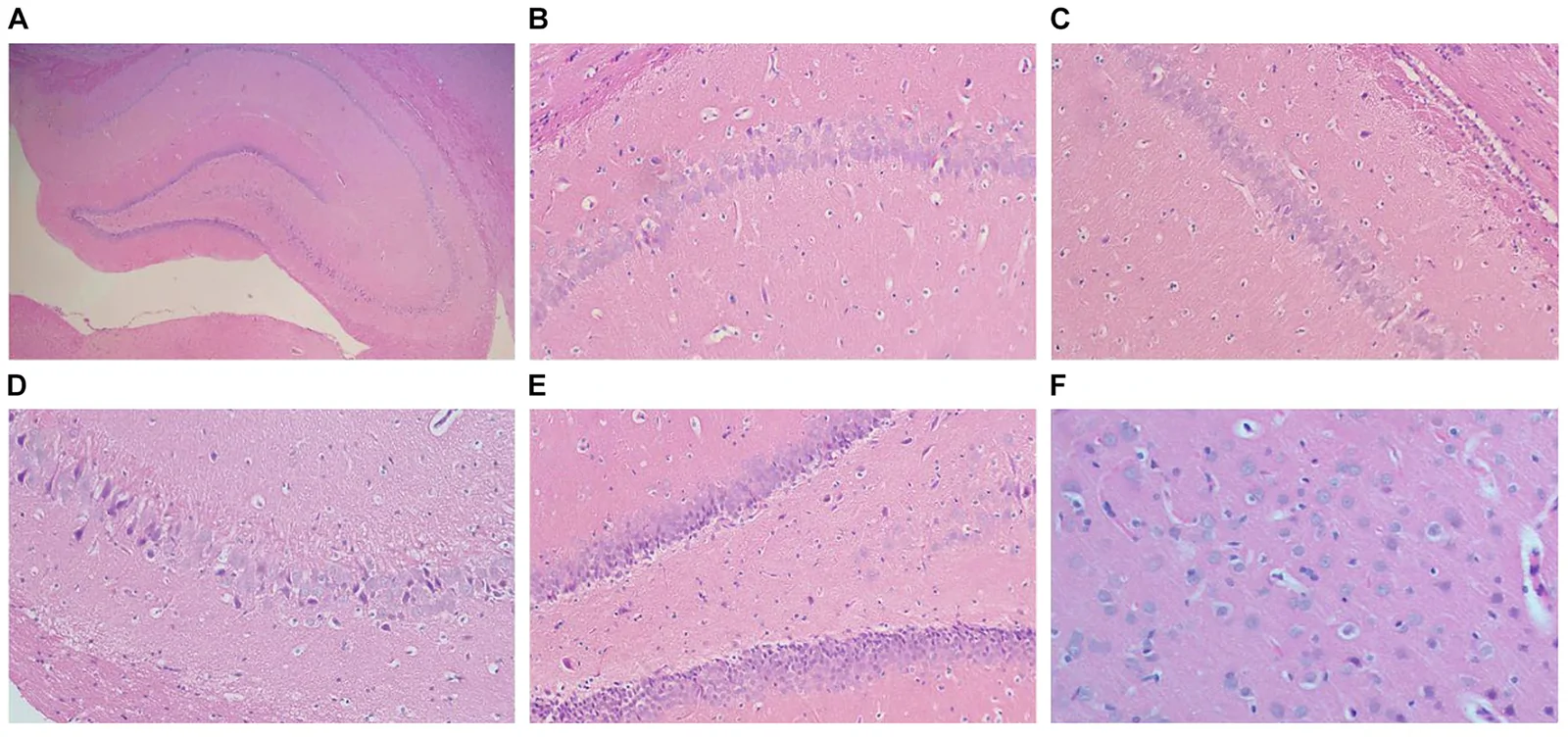
Representative H&E-stained images of the C group. (A) Hippocampus, C-shaped appearance at low magnification (40×). (B–E) Normal neuronal and glial architecture in hippocampal subregions CA1–CA3 and dentate gyrus (200×). (F) Normal nuclear and cytoplasmic features in cortical neurons and glial cells (400×).

**Figure 5:**
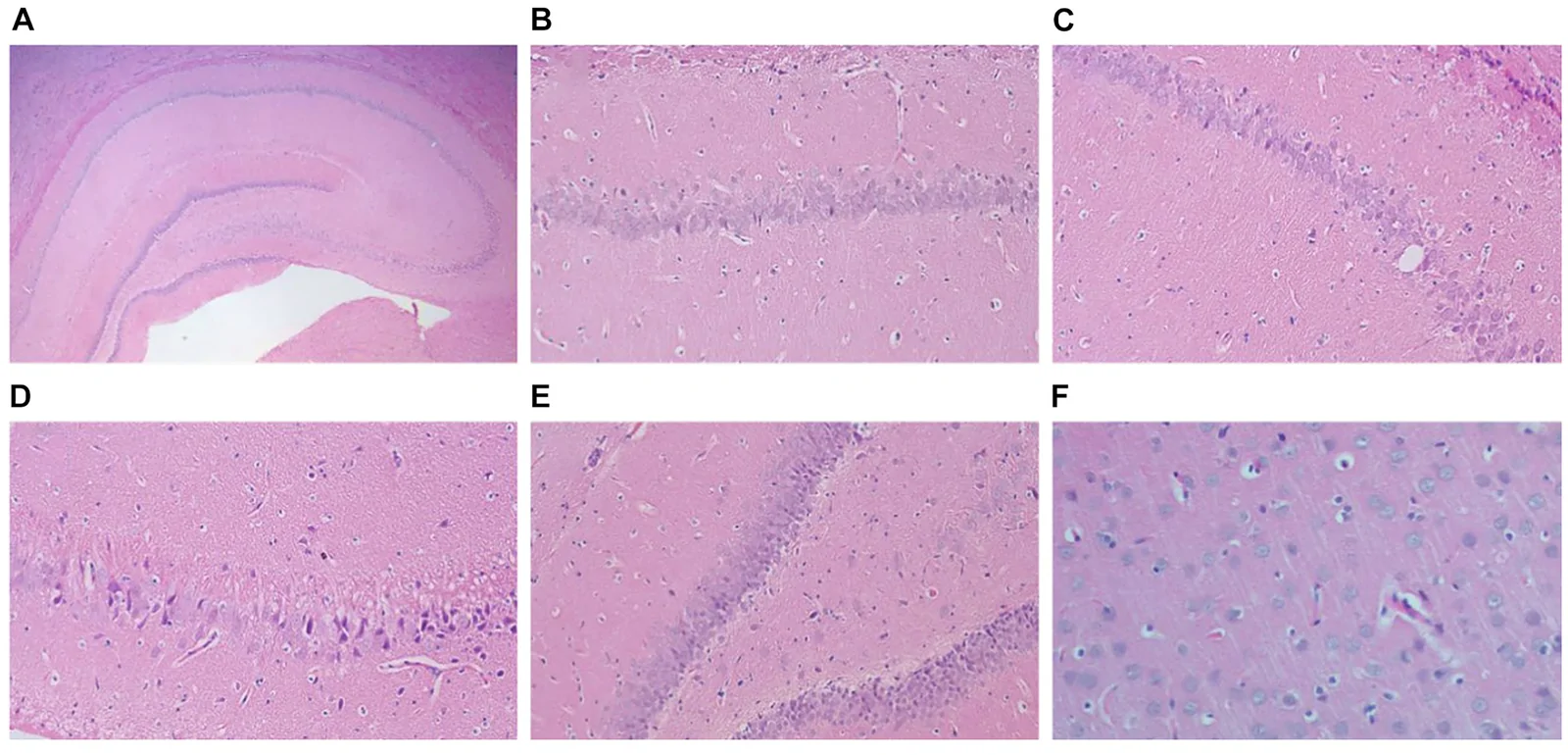
Representative light microscopic images with H&E staining of the SF group. At low magnification (A) (40×), the hippocampus displays its typical C-shaped structure. Neurons and neuroglia in the CA1 region (B), CA2 region (C), CA3 region (D), and dentate gyrus (E) exhibit normal morphology and distribution (200×). The nuclei and cytoplasmic features of neurons and glial cells in the cerebral cortex also appear normal (F) (400×).

In parasagittal sections of the hippocampi from the AD group, the hippocampal size was markedly reduced. Most pyramidal cell bodies in the CA regions appeared irregular and loosely arranged. Numerous degenerated neurons with dark, shrunken, and pyknotic nuclei were observed. Some neurons showed intracytoplasmic vacuolization, while intercellular spaces revealed edema. Glial cells displayed increased nuclear chromatin and cytoplasmic vacuolization (Figs. 6A–6E). Furthermore, condensed intracellular structures (neurofibrillary tangles (NFTs)-like) were present in certain neurons (Fig. 6H).

**Figure 6:**
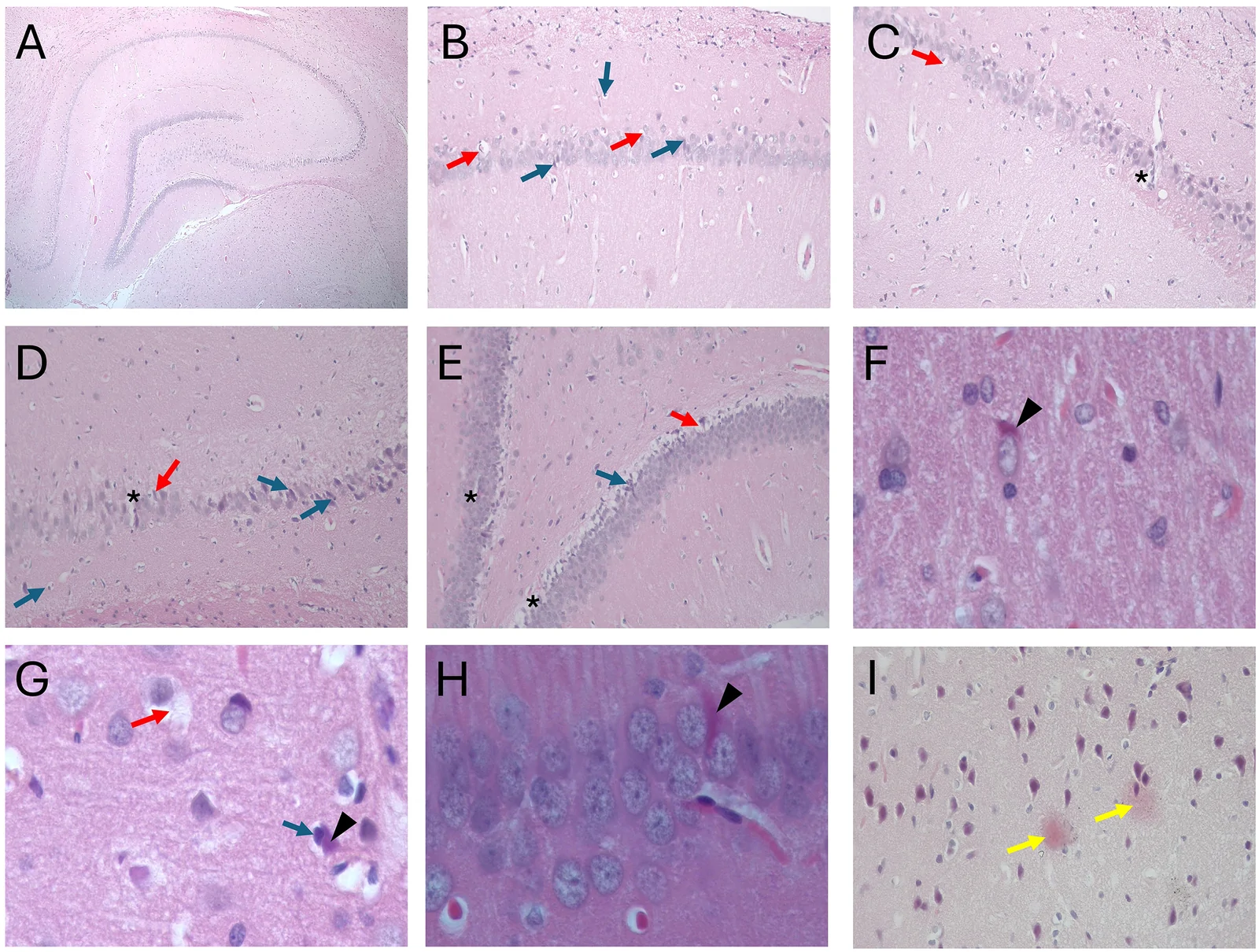
Representative light microscopic images with H&E staining of the AD group. At low magnification (A) (40×), the hippocampus shows its characteristic C-shaped structure. In the CA1 region (B), CA2 region (C), CA3 region (D), and dentate gyrus (E), neurons and neuroglial cells exhibited heterochromia, pyknosis, cytoplasmic shrinkage (blue arrow), and intracytoplasmic vacuolization (red arrow). Edematous areas and localized degeneration were evident in the intercellular spaces (*) (200×). In the cerebral cortex, some neurons showed nuclear chromatin condensation (blue arrow), cytoplasmic vacuolization (red arrow), and irregular NFTs (arrowhead) (F, G) (1,000×). Neurofibrillary condensation was also present in hippocampal neurons (H) (1,000×). Senile plaque-like structures of various diameters were identified in the cortex (yellow arrow) (I) (400×).

In the cerebral cortex, increased nuclear chromatin, cytoplasmic vacuolization, and condensed intracellular structures were detected in some neurons (Fig. 6G). Additionally, structures resembling extracellular plaque-like deposits of varying diameters were identified in the cortex (Fig. 6I).

In parasagittal sections of the AD + LE group hippocampi, degenerated neurons with pyknotic nuclei and intracytoplasmic vacuolization were observed, though in fewer numbers than in the AD group. Edema was present in the intercellular regions, and a small number of glial cells displayed increased nuclear chromatin and cytoplasmic vacuolization (Figs. 7A–7E). In the cerebral cortex, although some neurons exhibited chromatin condensation and cytoplasmic vacuolization, their occurrence was infrequent (Fig. 7F).

**Figure 7:**
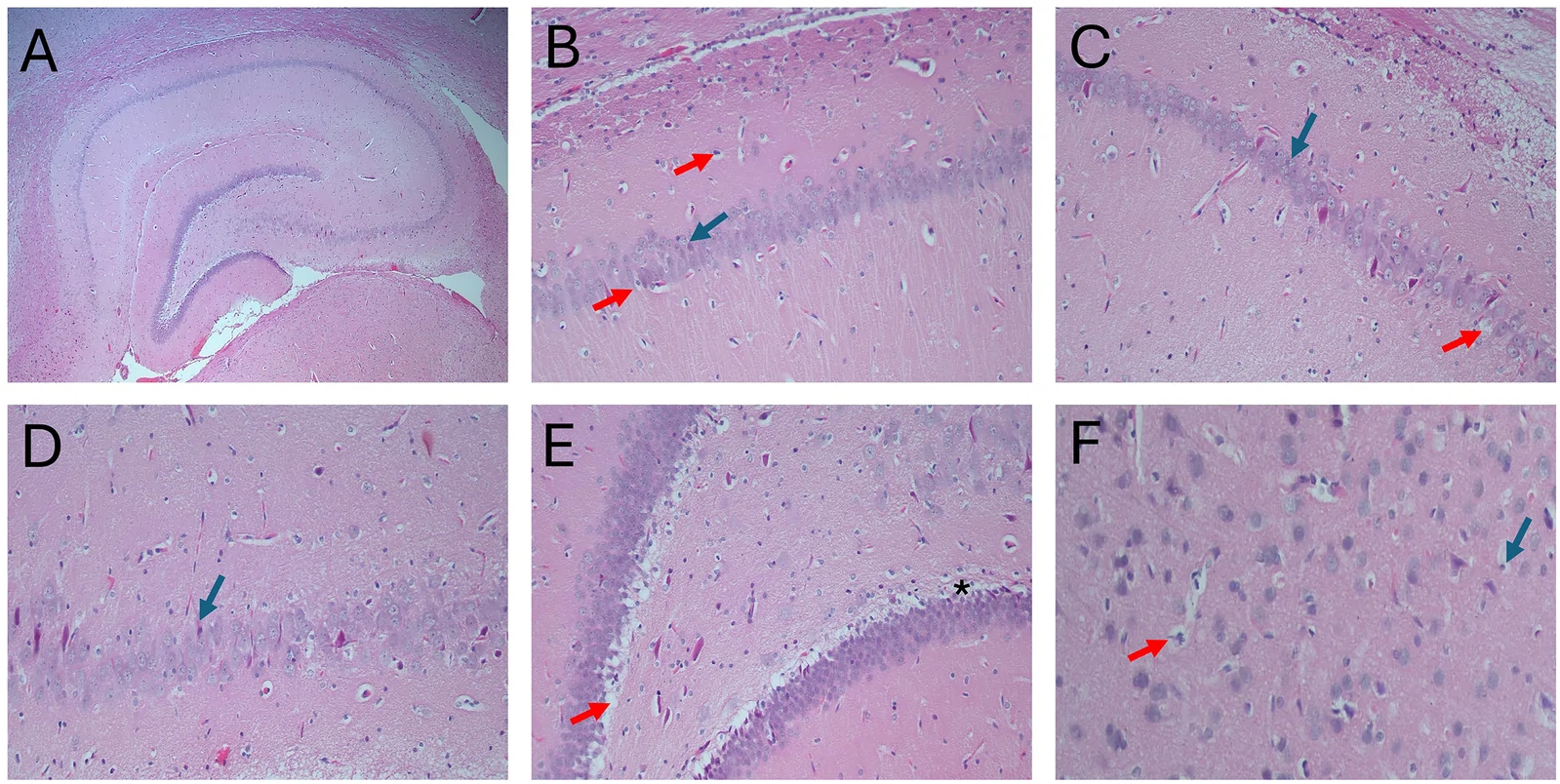
Representative light microscopic images with H&E staining of the AD + LE group. At low magnification (A) (40×), the hippocampus retains its C-shaped structure. In the CA1 (B), CA2 (C), CA3 (D), and dentate gyrus (E) regions, a small number of neurons and neuroglial cells exhibited heterochromia, pyknosis, cytoplasmic shrinkage (blue arrow), and intracytoplasmic vacuolization (red arrow). Intercellular spaces contained edematous areas and occasional degeneration (*) (200×). In the cerebral cortex, a limited number of cells demonstrated intracytoplasmic vacuolization (red arrow) and pyknosis (blue arrow) (F) (400×).

In parasagittal sections of the AD + HE group hippocampi, fewer degenerated neurons with pyknotic nuclei and intracytoplasmic vacuolization were observed compared with the AD + LE group. A small number of glial cells showed increased nuclear chromatin and cytoplasmic vacuolization (Figs. 8A–8E). In the cerebral cortex, some neurons exhibited increased nuclear chromatin, though cytoplasmic vacuolization was minimal (Fig. 8F).

**Figure 8:**
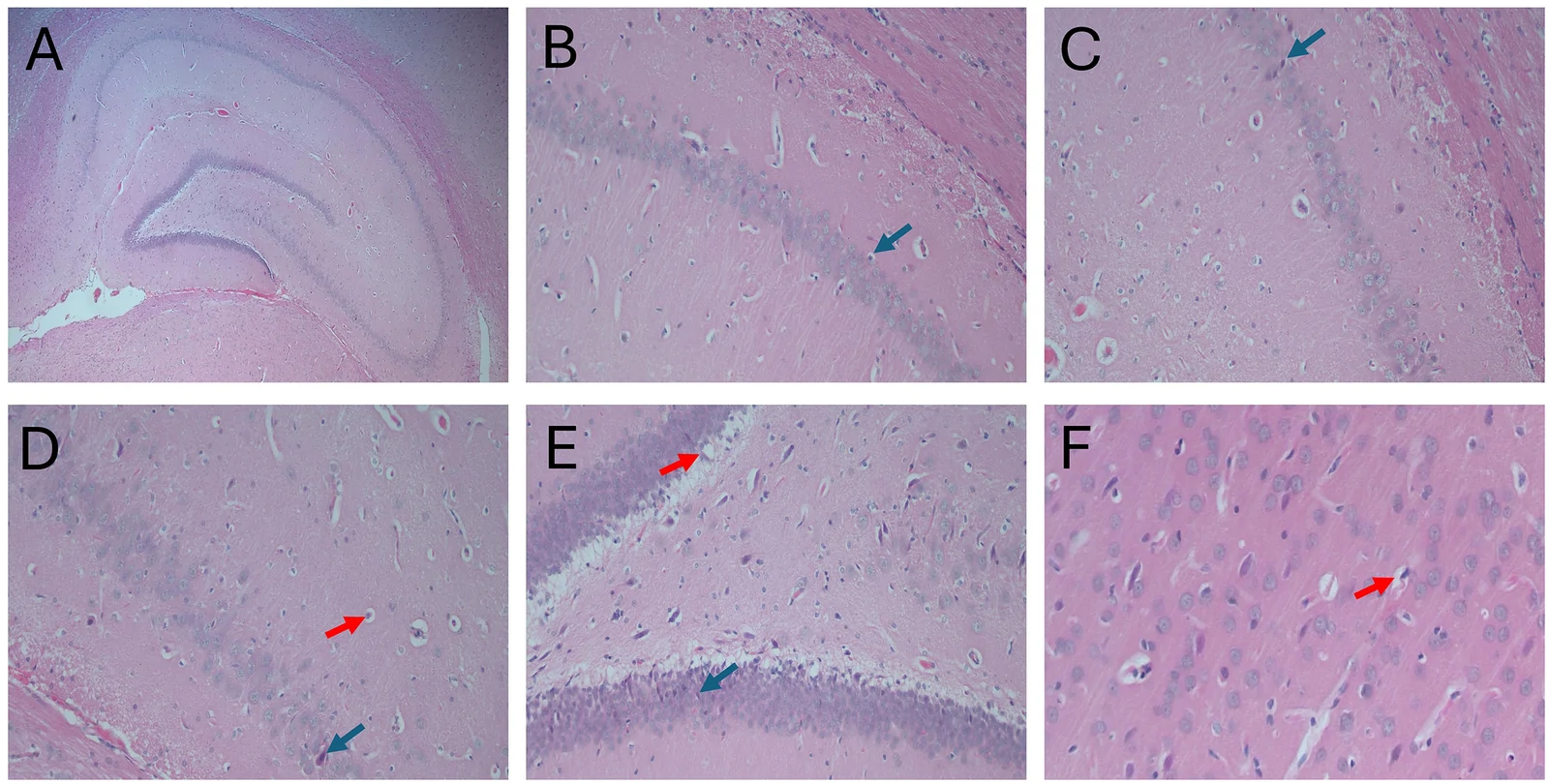
Representative light microscopic images with H&E staining of the AD + HE group. At low magnification (A) (40×), the hippocampus shows its typical C-shape. A small number of neurons in the CA1 (B), CA2 (C), CA3 (D), and dentate gyrus (E) regions demonstrated pyknosis (blue arrow) and intracytoplasmic vacuolization (red arrow) (200×). In the cerebral cortex, only a few cells displayed intracytoplasmic vacuolization (red arrow), and degeneration was minimal (F) (400×).

Qualitative evaluation of H&E-stained hippocampal sections indicated an apparent attenuation of histopathological alterations in the treatment groups compared with the AD group.

### Immunohistochemical findings

Serial sections (10 per subject) were prepared from paraffin blocks at defined intervals in the hippocampal region. In immunohistochemically stained sections, the immunopositivity rates of p-Tau and APP antibodies in the CA1, CA2, CA3, and dentate gyrus (DG) regions were assessed at 200× magnification. Mean values for each region per subject were calculated, and overall uptake rates were expressed as percentages using the ImageJ program.

When p-Tau immunopositivity in the hippocampus was compared across groups (Figs. 9, 10 and Table 2), no statistically significant difference were observed between the C and SF groups (*p* = 1.000). In contrast, the AD, AD + LE, and AD + HE groups showed significantly higher rates compared with the C and SF groups. Although immunopositivity in the AD + LE and AD + HE groups was lower than in the AD group, the decrease was not statistically significant (*p* = 0.108 and 0.118, respectively). Similarly, the AD + HE group values were lower than those of the AD + LE group, but this reduction was not significant (*p* = 1.000).

**Figure 9:**
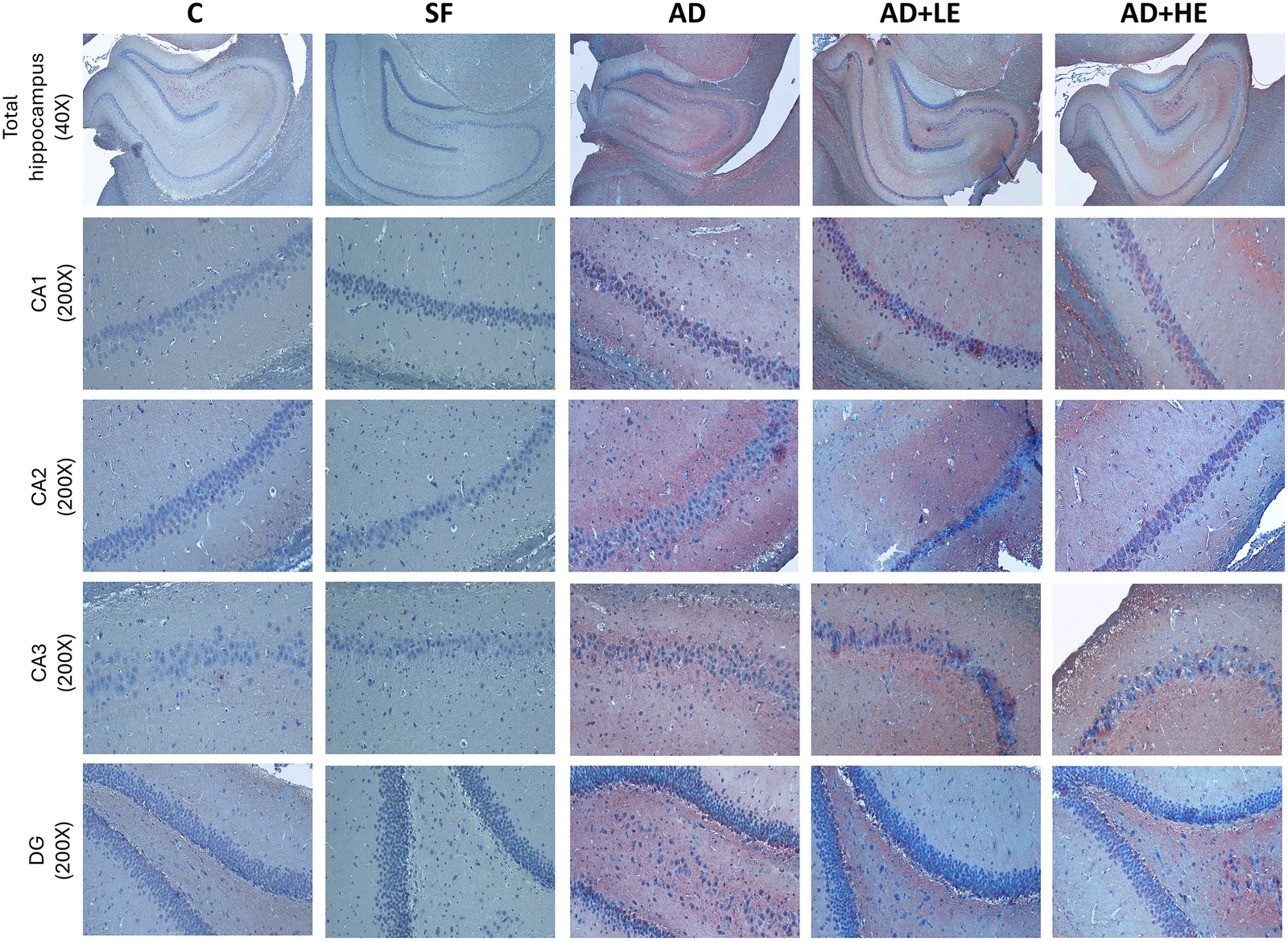
Representative images from anti-p-Tau immunohistochemical experiments in hippocampal sections from all groups (AEC & hematoxylin).

**Figure 10:**
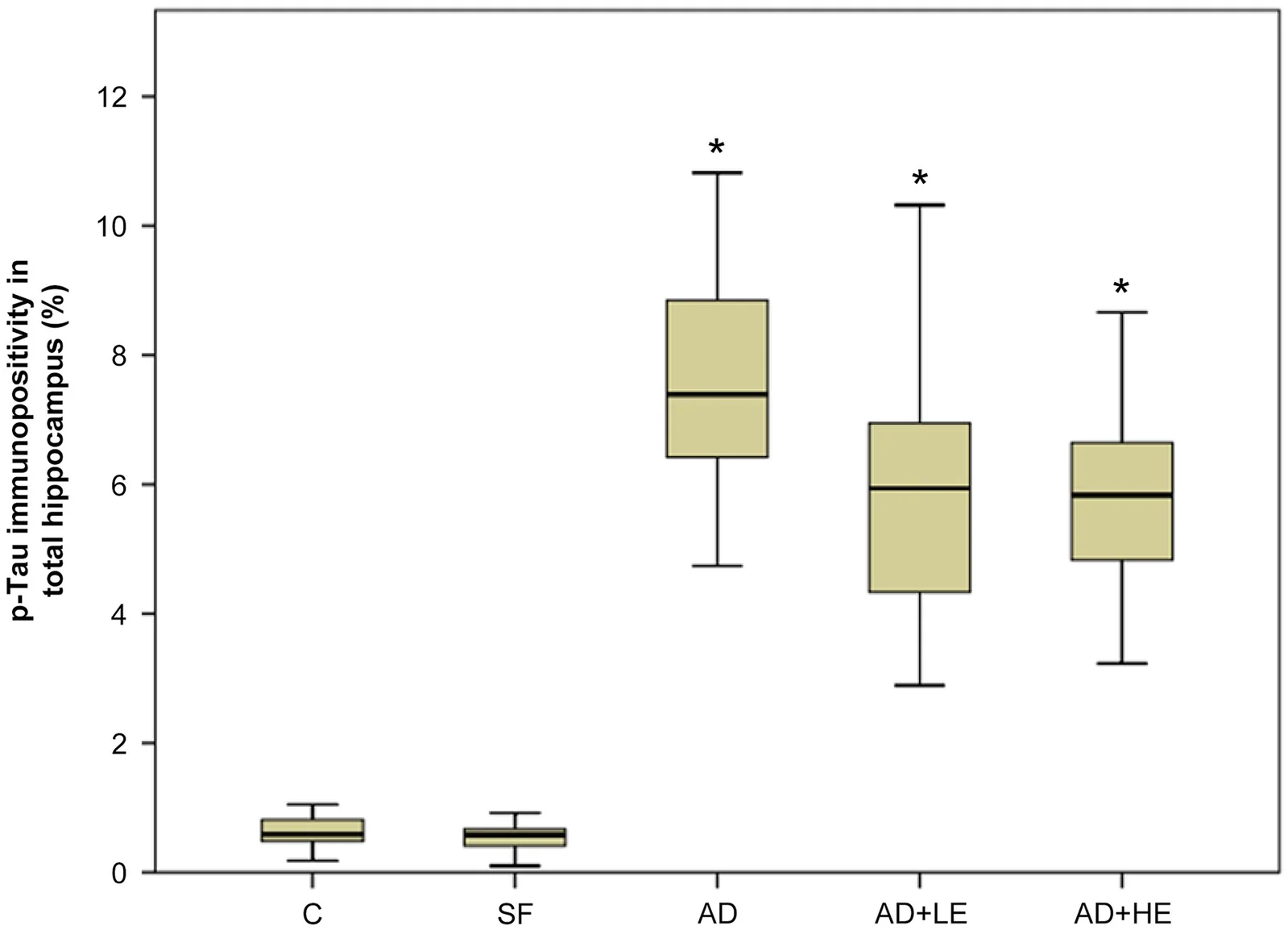
Total anti p-Tau immunopositivity rates in hippocampus sections of all groups. *p < 0.05 indicates a statistically significant difference between the compared groups.

**Table 2:** Descriptive data of anti p-Tau and anti APP immunohistochemical analyses of all groups.

		p-Tau immunopositivity (%)		APP immunopositivity (%)	
		Mean ± *SD*	Median (min–max)	Mean ± *SD*	Median (min–max)
Total	C	0.62 ± 0.23	0.59 (0.18–1.05)	0.89 ± 0.32	0.90 (0.36–1.66)
	SF	0.54 ± 0.19	0.58 (0.10–0.92)	1.05 ± 0.32	1.06 (0.51–1.73)
	AD	7.59 ± 1.64	7.39 (4.74–10.82)	9.45 ± 1.99	8.87 (6.48–13.21)
	AD + LE	5.93 ± 1.73	5.94 (2.89–10.32)	8.42 ± 1.15	8.31 (5.98–10.62)
	AD + HE	5.94 ± 1.33	5.84 (2.23–8.66)	6.19 ± 1.12	5.84 (4.68–9.07)
CA1	C	0.42 ± 0.54	0.13 (0.02–1.93)	0.21 ± 0.26	0.06 (0.01–1.06)
	SF	0.35 ± 0.38	0.18 (0.03–1.24)	0.28 ± 0.38	0.11 (0.01–1.80)
	AD	8.77 ± 4.33	8.50 (3.31–22.48)	7.74 ± 2.91	7.85 (3.09–13.69)
	AD + LE	8.01 ± 2.56	8.02 (3.65–15.64)	9.74 ± 2.14	9.84 (5.63–15.30)
	AD + HE	6.76 ± 2.23	6.66 (2.09–10.58)	6.03 ± 2.14	5.39 (3.06–12.64)
CA2	C	0.34 ± 0.48	0.09 (0.00–2.04)	1.11 ± 0.88	1.10 (0.04–3.61)
	SF	0.31 ± 0.42	0.12 (0.01–1.42)	1.08 ± 0.57	1.14 (0.02–2.06)
	AD	11.08 ± 4.30	9.07 (3.77–19.39)	7.42 ± 2.16	7.57 (4.06–12.65)
	AD + LE	5.32 ± 2.77	4.29 (1.64–13.28)	6.41 ± 2.28	5.91 (3.09–11.36)
	AD + HE	3.02 ± 1.54	3.22 (0.14–5.49)	5.64 ± 1.84	5.36 (3.07–10.45)
CA3	C	0.38 ± 0.40	0.21 (0.01–1.50)	0.64 ± 0.59	0.55 (0.01–1.91)
	SF	0.33 ± 0.33	0.18 (0.01–1.06)	1.05 ± 0.73	0.95 (0.03–2.95)
	AD	6.83 ± 1.92	6.57 (3.56–11.25)	9.36 ± 2.68	9.87 (4.69–14.91)
	AD + LE	5.77 ± 2.24	5.08 (3.06–11.28)	6.99 ± 2.48	7.13 (3.08–11.29)
	AD + HE	5.54 ± 2.69	4.78 (1.60–14.36)	7.59 ± 1.86	7.73 (3.55–11.28)
DG	C	0.73 ± 0.55	0.58 (0.01–1.94)	1.61 ± 0.77	1.58 (0.06–3.12)
	SF	0.67 ± 0.49	0.59 (0.03–1.69)	1.78 ± 0.52	1.68 (1.08–3.21)
	AD	8.61 ± 2.96	8.36 (4.36–17.36)	13.29 ± 5.30	11.27 (5.31–24.61)
	AD + LE	4.61 ± 2.08	4.17 (1.04–9.15)	10.54 ± 2.76	10.65 (4.09–14.61)
	AD + HE	5.19 ± 2.42	4.49 (2.05–10.64)	5.51 ± 1.63	5.23 (3.51–9.63)

When hippocampal subregions were analyzed separately (Figs. 9, 11 and Table 2), all regions (CA1, CA2, CA3, and DG) showed a significant increase in immunopositivity in the AD, AD + LE, and AD + HE groups compared with the C and SF groups. In the CA1 and CA3 regions, immunopositivity in the AD + LE and AD + HE groups decreased relative to the AD group, but the differences were not significant. Values in the AD + HE group were also lower than those of the AD + LE group, but again, this difference was not significant (*p* = 1.000). Notably, in the CA2 and DG regions, immunopositivity was significantly reduced in both the AD + LE and AD + HE groups compared with the AD group (CA2: *p* = 0.019 and *p* = 0.017; DG: *p* = 0.002 and 0.020, respectively). However, no significant differences were observed between the AD + LE and AD + HE groups for any hippocampal region (CA1, CA2, CA3, or DG; *p* = 1.000, 0.365, 1.000, and 1.000, respectively).

**Figure 11:**
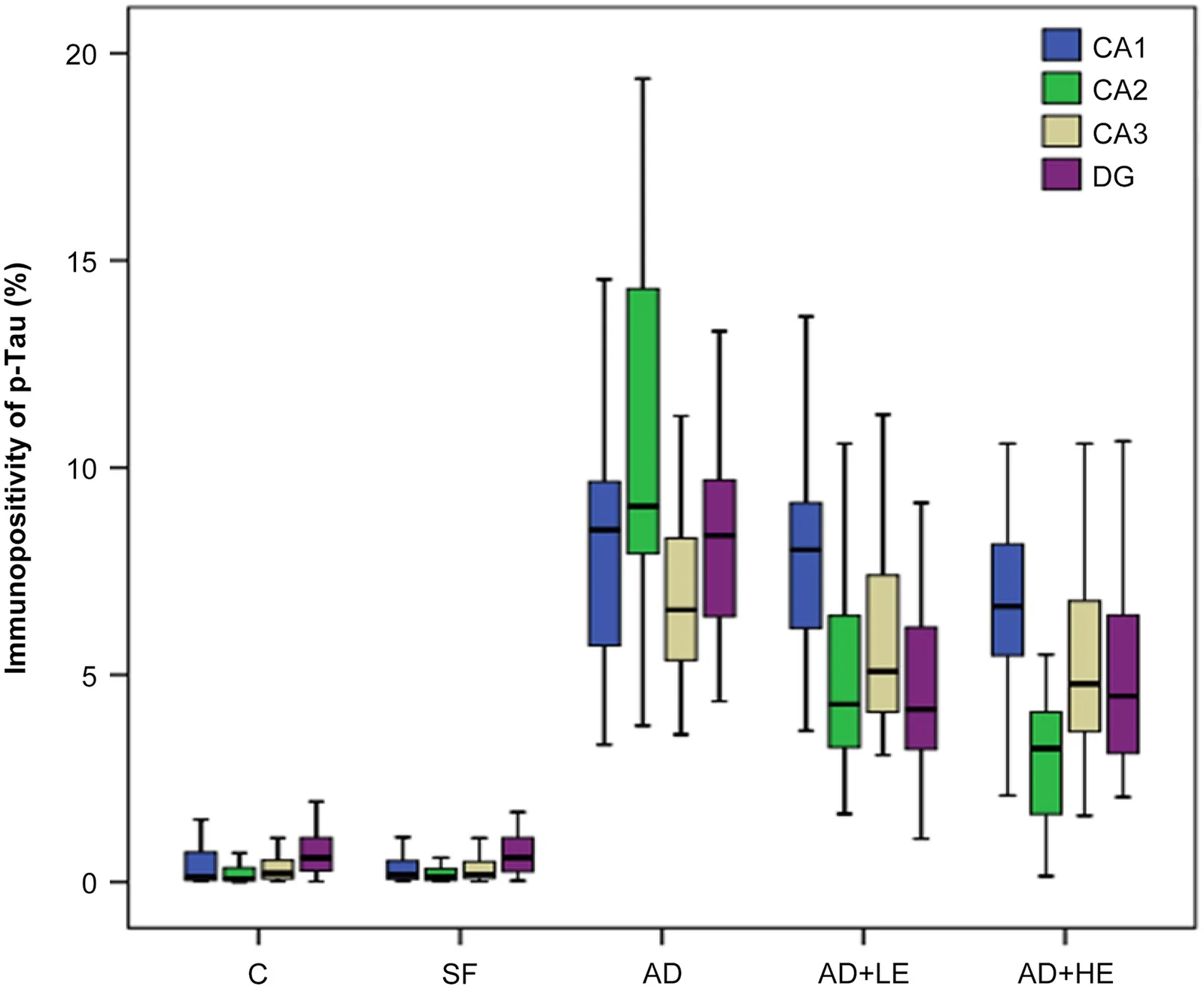
Regional anti p-Tau immunopositivity rates in hippocampus sections of all groups.

When APP immunopositivity in the total hippocampus was evaluated across groups (Figs. 12, 13 and Table 2), no statistically significant difference was observed between the C and SF groups (*p* = 1.000). In contrast, the AD, AD + LE, and AD + HE groups demonstrated a statistically significant increase compared with the C and SF groups. Although the uptake rates in the AD + LE group were lower than those in the AD group, this decrease was not statistically significant (*p* = 1.000). By contrast, the AD + HE group showed a significant reduction in immunopositivity compared with the AD group. Moreover, values in the AD + HE group were significantly lower than those of the AD + LE group (*p* = 0.006).

**Figure 12:**
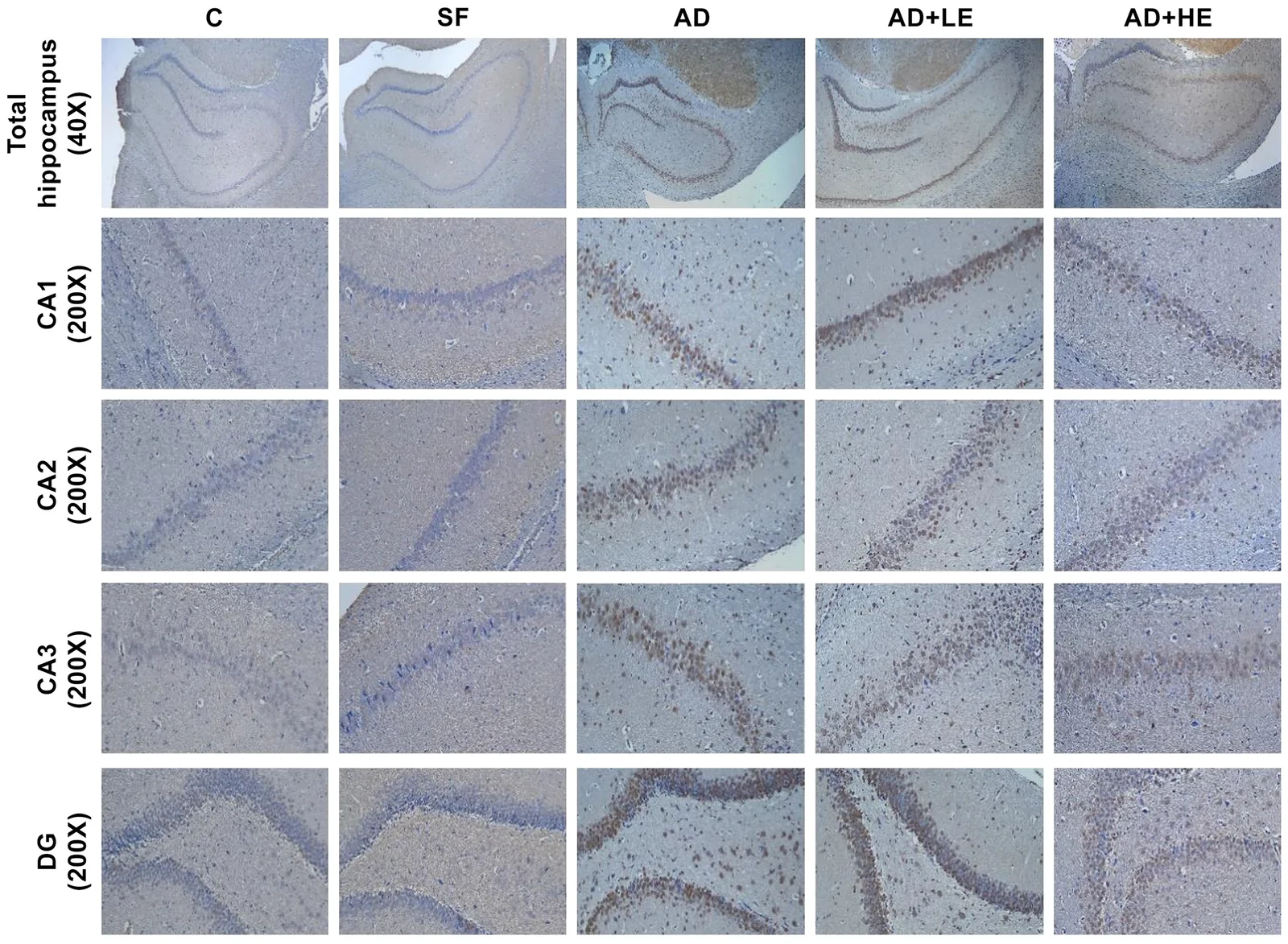
Representative images from anti-APP immunohistochemical experiments in hippocampal sections from all groups (DAB & hematoxylin).

**Figure 13:**
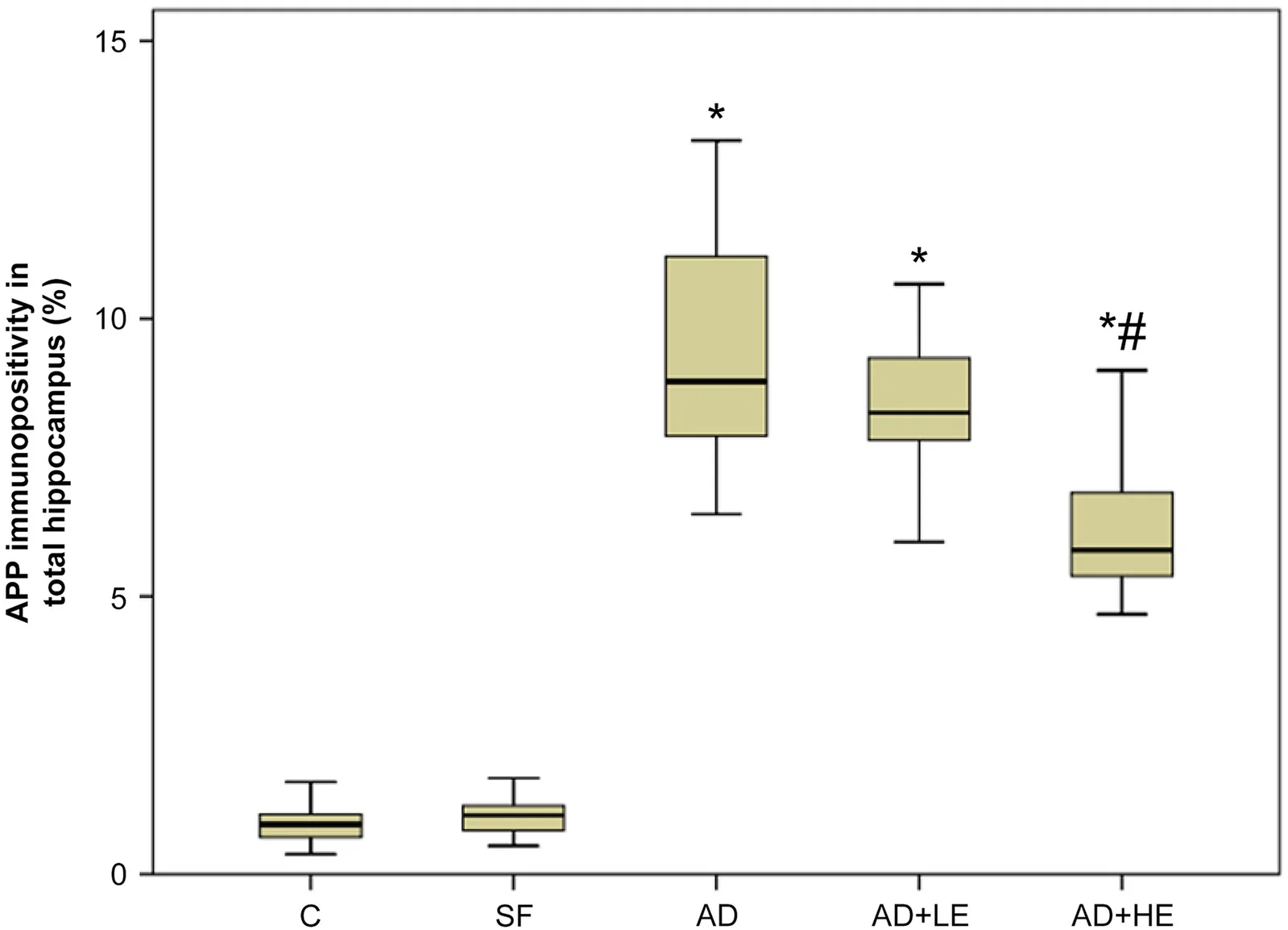
Total anti-APP immunopositivity rates in hippocampal sections of all groups. *p < 0.05 and ^#^*p* < 0.01 indicate statistically significant differences between the compared groups.

When hippocampal subregions were assessed separately (Figs. 12, 14 and Table 2), all regions (CA1, CA2, CA3, and DG) in the AD, AD + LE, and AD + HE groups exhibited significantly higher immunopositivity compared with the C and SF groups. Comparison of the AD and AD + LE groups revealed no significant differences across all regions. In contrast, when the AD and AD + HE groups were compared, values were reduced in the AD + HE group, with a statistically significant decrease observed only in the DG region. Furthermore, comparison of the AD + LE and AD + HE groups revealed significant reductions in the CA1 and DG regions in the AD + HE group (*p* = 0.004 and 0.002, respectively).

**Figure 14:**
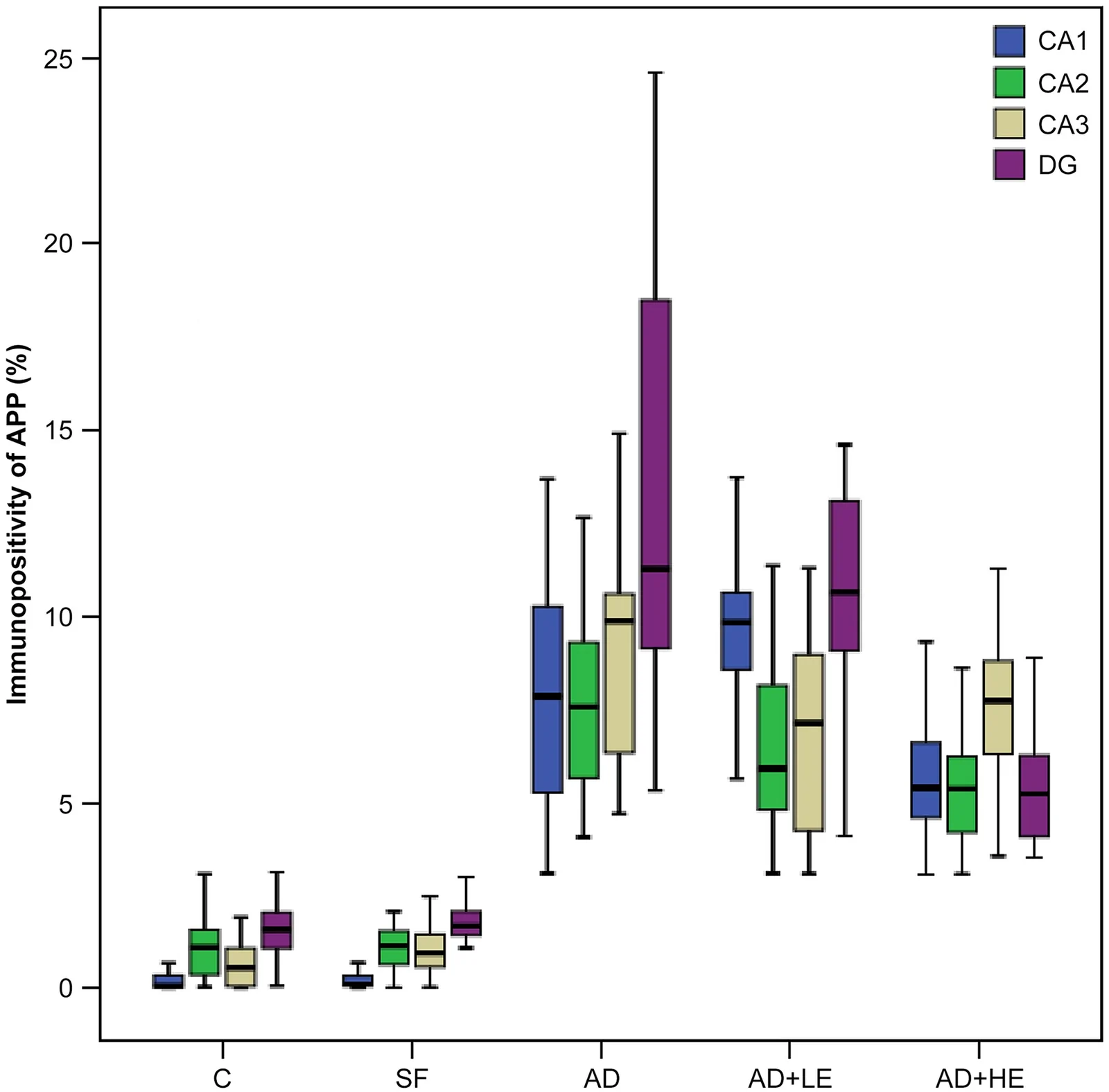
Regional anti APP immunopositivity rates in hippocampus sections of all groups.

The full set of pairwise group comparisons for p-Tau and APP immunoreactivity across the total hippocampus, CA1, CA2, CA3, and DG regions is provided in Table 3, with values considered statistically significant according to Bonferroni-corrected Mann–Whitney U testing highlighted in bold.

**Table 3:** Pairwise comparisons of groups. Values considered statistically significant as a result of the Bonferroni corrected Mann–Whitney test are highlighted in bold.

Compared groups	p-Tau					APP				
	Total	CA1	CA2	CA3	DG	Total	CA1	CA2	CA3	DG
C-SF	1.000	1.000	1.000	1.000	1.000	1.000	1.000	1.000	1.000	1.000
C-AD	**<0.001**	**<0.001**	**<0.001**	**<0.001**	**<0.001**	**<0.001**	**<0.001**	**<0.001**	**<0.001**	**<0.001**
C-AD + LE	**<0.001**	**<0.001**	**<0.001**	**<0.001**	**<0.001**	**<0.001**	**<0.001**	**<0.001**	**<0.001**	**<0.001**
C-AD + HE	**<0.001**	**<0.001**	**<0.001**	**<0.001**	**<0.001**	**<0.001**	**<0.001**	**<0.001**	**<0.001**	**<0.001**
SF-AD	**<0.001**	**<0.001**	**<0.001**	**<0.001**	**<0.001**	**<0.001**	**<0.001**	**<0.001**	**<0.001**	**<0.001**
SF-AD + LE	**<0.001**	**<0.001**	**<0.001**	**<0.001**	**<0.001**	**<0.001**	**<0.001**	**<0.001**	**<0.001**	**<0.001**
SF-AD + HE	**<0.001**	**<0.001**	**<0.001**	**<0.001**	**<0.001**	**<0.001**	**<0.001**	**<0.001**	**<0.001**	**<0.001**
AD-AD + LE	0.108	1.000	**0.019**	1.000	**0.002**	1.000	0.557	1.000	0.183	1.000
AD-AD + HE	0.118	1.000	**<0.001**	0.595	**0.020**	**<0.001**	1.000	0.297	0.641	**<0.001**
AD + LE-AD + HE	1.000	1.000	0.365	1.000	1.000	**0.006**	**0.004**	1.000	1.000	**0.002**

## Discussion

The main finding of this exploratory study is that oral CELN administration was associated with partial changes in selected FDG-PET metabolic parameters and region-specific reductions in hippocampal p-Tau and APP immunoreactivity in a fibrillar Aβ_1–42_-induced neurodegenerative rat model. These findings should be interpreted as preliminary biomarker-level observations rather than evidence of behavioral improvement, disease modification, or established clinical efficacy.

The blood–brain barrier (BBB) protects the complex and dynamic structure of the central nervous system but also presents a significant obstacle to intracerebral drug delivery. Consequently, large-molecule drugs cannot cross the BBB, and many small-molecule drugs cross only partially. One commonly employed strategy is to load drugs into carriers capable of traversing the BBB (Zhong et al., 2023). Exosomes show greater efficiency in intracellular uptake than traditional carriers such as liposomes or nanoparticles due to their structural similarity to the cell membrane, with sizes ranging from 30 to 100 nm. Their small size, ability to penetrate deep tissues, low zeta potential during long-term circulation, and membrane-like structure provide distinct advantages. Moreover, some exosomes exhibit enhanced resistance to immune system clearance (Khatoon, Alam & Sharma, 2021; Vader et al., 2016). Morphological changes such as basement membrane thickening and reduced micro vessel density, observed postmortem in the brains of patients with Alzheimer’s disease and in animal models, indicate BBB damage (Claudio, 1996; Halliday et al., 2015; Sweeney, Sagare & Zlokovic, 2018). *In vitro* studies have also shown pathological changes in the blood-brain barrier with Alzheimer’s disease (Gustafsson et al., 2018; Qosa et al., 2014). In Alzheimer’s disease, BBB impairment accelerates pathological processes that damage brain tissue. Increased BBB permeability may affect the delivery of small molecules to the brain. Exosomes, because of their membrane-like structure and small size, may improve the transport of therapeutic compounds to the brain by promoting more effective intracellular uptake than conventional carriers. In addition, their reduced susceptibility to immune-mediated clearance allows exosomes to remain stable in long-term circulation, potentially enhancing their therapeutic effectiveness The present study does not provide direct evidence that orally administered CELNs crossed the blood–brain barrier or accumulated in brain tissue. Therefore, the observed FDG-PET and immunohistochemical changes cannot be used to infer a specific brain biodistribution mechanism. Future studies using labeled CELNs, biodistribution assays, and gut–brain axis-focused designs are required to determine whether the observed effects are mediated by direct brain delivery, peripheral immune/metabolic modulation, or indirect gut–brain signaling.

A limitation of this study is that further research is required to clarify how CELNs affect the BBB in a fibrillar Aβ_1–42_-induced neurodegenerative rat model. Future studies comparing the miRNA content of CELNs with miRNA profiles in brain tissue and examining their potential in a fibrillar Aβ_1–42_-induced neurodegenerative rat model and other neurological diseases may help address these limitations. Meanwhile, studies of mesenchymal stem cell (MSC)-derived exosome-based approaches show promise in reducing Aβ_1–42_ and tau pathology, suppressing neuroinflammation, and improving cognitive function by targeting Alzheimer’s disease pathogenesis (Cui et al., 2019; Qi et al., 2020).

The Aβ_1–42_ preparation was not directly characterized before injection. The 37 °C/7-day incubation protocol is expected to enrich fibrillar Aβ species; therefore, the findings should not be generalized to soluble oligomer-driven AD pathology. The Aβ_1–42_-induced model represents an acute neuroinflammatory and neurodegenerative phenotype, which is suitable for investigating early therapeutic interventions targeting metabolic and proteinopathic changes, but it does not fully recapitulate the chronic, age-dependent progression of human AD. The model should be interpreted as a short-term fibrillar Aβ_1–42_-induced neurodegeneration/neuroinflammation model. A limitation of this study is the absence of behavioral data. Our study focused on histochemical and micro-PET imaging characterization of the fibrillar Aβ_1–42_-induced neurodegenerative rat model. While these complementary approaches provide evidence of neuropathological and metabolic alterations, they do not assess cognitive or behavioral outcomes. Therefore, the findings are limited to the molecular and metabolic level and cannot be extended to cognitive deficits. Future studies should incorporate cubicle-based histological quantification and behavioral assays targeting spatial learning and memory—including Barnes maze, Y-maze, Morris water maze, or novel object recognition testing—to determine whether the observed molecular improvements translate into functional cognitive benefits.

MicroPET/CT imaging has become a valuable tool for investigating AD pathology in animal models. Several studies have demonstrated the effectiveness of PET tracers in detecting Aβ_1–42_ accumulation. For example, the [^18^F] O-FEt-PIB tracer showed increased binding in the hippocampus and thalamus of Aβ-injected rats (Zheng et al., 2008). Similarly, [F-18] FDDNP exhibited progressive binding associated with age-dependent Aβ accumulation in the hippocampus and frontal cortex of transgenic rat models. The specificity of [F-18] FDDNP was confirmed through naproxen blockade and anti-Aβ antibody treatment (Kepe et al., 2005; Teng et al., 2011). Pharmacological studies revealed that [F-18] FDDNP has high binding affinity for both Aβ aggregates and NFTs (tau). The significant reduction in tracer binding after naproxen competition and anti-Aβ antibody administration further confirmed its target specificity. These findings suggest that [F-18] FDDNP-based PET imaging offers a reliable *in vivo* method for assessing amyloid pathology in Alzheimer’s disease. Furthermore, intrahippocampal injection of Aβ_1–42_ has been proposed as a short-term model for examining the effects of Aβ_1–42_ in specific brain regions (Facchinetti, Bronzuoli & Scuderi, 2018).

MicroPET/CT imaging also plays an important role in investigating neurodynamic processes associated with brain injury. For instance, Zhang et al. (2015), reported significant glucose hypometabolism in the hippocampus of rats following pilocarpine-induced status epilepticus, which was associated with neuronal loss and astrogliosis. These results underscore the utility of microPET/CT in studying both the progression of brain injury and underlying neuropathological changes (Zhang et al., 2015).

The glucose hypometabolism observed in the AD group, reflected by reduced SUV values (Fig. 2), is consistent with FDG-PET findings reported in experimental and clinical AD-related contexts. This metabolic impairment supports the presence of brain-level dysfunction in the fibrillar Aβ_1–42_-induced model, but it should not be interpreted as a complete validation of the human Alzheimer’s disease phenotype. Our findings indicate partial treatment-associated changes in SUV values, with the most evident difference observed in SUVmax in the AD + HE group (Figs. 2A, 2B). These results suggest that CELN administration was associated with selected neurometabolic differences, particularly in SUVmax. However, SUVmean did not demonstrate a statistically significant difference between AD and AD + HE groups, and the present data do not establish full reversal of metabolic alterations or behavioral improvement. The observation that SUVmax, but not SUVmean, reached significance in the high-dose group may reflect the timing of PET imaging, which was performed immediately after the 14-day treatment regimen when circulating CELN levels may have begun to decline. Future pharmacokinetic studies assessing CELN persistence and biodistribution should incorporate pixel- or voxel-based quantitative PET analyses to support SUV findings, thereby helping optimize dosing schedules and determine whether earlier imaging time points yield more robust dose-response relationships.

Previous studies have shown that Aβ accumulation in Alzheimer’s disease animal models leads to ultrastructural deterioration and organelle damage (Upadhaya et al., 2012). The pathological development of Alzheimer’s disease remains complex and incompletely understood. Microscopically, disease progression is characterized by the accumulation of Aβ and tau proteins, which aggregate into Aβ plaques and NFTs, respectively (Gallardo & Holtzman, 2019; Ribeiro, Lopes & Gomes, 2023; Yuyama et al., 2019). Pathological hallmarks such as NFTs and Aβ typically appear first in the entorhinal cortex and hippocampus before spreading to areas such as the prefrontal cortex (Poulakis et al., 2018). Under normal conditions, APP cleavage produces Aβ peptides, which are rapidly cleared. In Alzheimer’s disease, however, Aβ monomers aggregate into oligomers and fibrils, leading to plaque formation and neuronal loss (Rajendran et al., 2006; Yuyama et al., 2019).

Yuyama et al. (2019) demonstrated that oral administration of plant-derived glucosylceramide (GlcCer) from *Amorphophallus konjac* in APP transgenic mice significantly reduced Aβ levels and plaque densities in hippocampal tissue. ELISA measurements confirmed significant reductions in Aβ40 and Aβ42 in the hippocampus and cerebral cortex compared with controls. Although histological evaluation revealed no significant differences between groups, immunohistochemistry showed markedly reduced Aβ reactivity and plaque density in the hippocampus (Yuyama et al., 2019). Similarly, exosome-like nanoparticles from *Momordica charantia* exhibited neuroprotective effects against ischemic brain injury by alleviating BBB damage and preventing neuronal apoptosis in rats (Cai et al., 2022). In another study, Silibinin-loaded macrophage-derived exosomes significantly reduced Aβ plaques in the hippocampus of Alzheimer’s disease rats, with greater cognitive improvement compared with unloaded exosome treatment, suggesting enhanced neuroprotection (Huo et al., 2021). A recent study further demonstrated the neuroprotective effects of PELNs in an Alzheimer’s disease cell model. Using Aβ-treated HT-22 mouse hippocampal neurons, researchers found that nanovesicles from *Lycium ruthenicum* Murray significantly reduced Aβ-induced apoptosis and oxidative stress. These effects were mediated through reductions in the Bax/Bcl-2 ratio and suppression of active Caspase-3 expression (Zhang et al., 2024).

To investigate a fibrillar Aβ_1–42_-induced neurodegeneration, we employed an Aβ_1–42_–induced rat model and examined hippocampal and prefrontal cortex alterations using light microscopy. In the AD group, cells in the hippocampal CA and DG regions appeared irregularly and loosely arranged, with evident neurofibrillary condensation, degeneration, and edema. Notably, these structural changes decreased in a dose-dependent manner in treatment groups that received CELNs. Apparent attenuation of these alterations was observed in treatment groups; however, these H&E findings were qualitative and were not supported by quantitative morphometry.

In this study, APP immunoreactivity, which increased significantly in the AD group compared to controls, was reduced in the total hippocampus after CELNs treatment. However, the reduction was statistically significant only in the high-dose CELNs group compared with the AD group. Regionally, APP uptake in the CA subfields showed no significant reduction, whereas a significant decrease was observed in the DG region following high-dose CELN treatment. APP immunostaining is not equivalent to direct Aβ_1–42_ quantification and should be interpreted only as an amyloid-pathway-associated marker. Future studies should include Aβ_1–42_-specific immunohistochemistry, ELISA, or biochemical quantification to evaluate peptide deposition directly.

Neurofibrillary tangles result from the accumulation and aggregation of abnormally phosphorylated tau protein (Liang et al., 2022). Elevated tau protein is a typical pathological feature of Alzheimer’s disease (Xin et al., 2021). Clinical and preclinical studies have shown that serum tau levels are higher in older adults and in individuals with cognitive impairment than in healthy controls. Both total tau (t-tau) and phosphorylated tau (p-tau) are commonly used biomarkers for the diagnosis of neurodegenerative diseases (Nam et al., 2020; Wang et al., 2022). Elevated t-tau levels have also been associated with smaller hippocampal volume, a relationship that persists throughout disease progression (Pase et al., 2019). In an experimental Alzheimer’s disease model, quercetin-loaded plasma exosomes demonstrated neuroprotective effects in okadaic acid-induced Alzheimer’s disease mice by inhibiting CDK5-mediated tau phosphorylation and reducing the formation of insoluble NFTs (Qi et al., 2020).

In our study, p-Tau immunoreactivity density increased significantly in the AD group compared with the control group. CELN treatment reduced p-Tau levels relative to the AD group, but this effect did not reach statistical significance in the CA1 and CA3 regions. Quantitative analysis revealed region-specific reductions in p-Tau immunoreactivity in treatment groups relative to the AD group. Nevertheless, comparisons between the AD+LE and AD+HE groups did not demonstrate a statistically confirmed dose-dependent effect.

It is important to emphasize that, given the absence of behavioral data and direct evidence of brain biodistribution, no conclusions can be drawn regarding the clinical or cognitive efficacy of CELNs. The findings presented here are strictly empirical observations at the molecular and metabolic level. The lack of specificity controls (vesicle-depleted coffee extract, caffeine-equivalent controls, and non-coffee nanoparticle controls) is a limitation. Consequently, the observed effects cannot be attributed specifically to CELNs. Future studies should include these controls to determine specificity and molecular characterization of plant-specific exosome markers such as TET8, PEN1, proteomics, lipidomics, caffeine/polyphenol quantification, and RNA cargo analyses to further confirm the identity of CELNs beyond size and morphological features. The absence of paired correlation between FDG-PET and IHC markers is a limitation; future studies should address this with appropriately powered and paired designs.

## Conclusions

In this exploratory fibrillar Aβ_1–42_-induced neurodegenerative rat model, oral CELN administration was associated with partial improvements in selected FDG-PET metabolic parameters and region-specific reductions in hippocampal p-Tau and APP immunoreactivity. However, these are strictly preliminary biomarker-level findings that support further investigation of CELNs; definitive confirmation of functional relevance requires additional behavioral, biodistribution, and mechanistic studies.

The study has limitations. The injected Aβ_1–42_ preparation was not directly characterized for aggregation state, behavioral testing was not performed, CELN brain biodistribution and cellular uptake were not assessed, Aβ_1–42_-specific immunostaining was not included, CELN molecular cargo composition was not analyzed, and paired animal-level correlation between FDG-PET and immunohistochemical endpoints was not performed. Therefore, the results should not be interpreted as evidence of behavioral efficacy, BBB penetration, direct brain delivery, or disease-modifying activity.

Future studies should include behavioral assays, labeled-CELN biodistribution studies, direct Aβ_1–42_ quantification, expanded molecular characterization of CELNs, additional specificity control groups, paired FDG-PET/IHC correlation analyses, and longer treatment periods to clarify mechanism, dose-response relationships, and functional relevance.

Together, these findings provide a preliminary basis for further preclinical investigation of coffee-derived exosome-like nanoparticles in neurodegenerative disease models.

## Supplemental Information

10.7717/peerj.21739/supp-1 Supplemental Information 1 ARRIVE 2.0 Checklist.

## Additional Information and Declarations

### Data Availability

The following information was supplied regarding data availability:

The data is available at Zenodo:

- Kucukbagriacik, Y., Saribas, G. S., Ates, K., Ozturk, C., Kahyaoglu, E., Kislal, S., Akgul, C. C., Karacalioglu, A. O., Goksoy, C.& Kantarcioglu, M. (2025). Therapeutic Potential of Coffee-Derived Exosome-Like Nanoparticles in an Experimental Aβ1–42-Induced Alzheimer’s Model: Evidence from FDG-PET Imaging and Tau/APP Expression [Dataset]. Zenodo. https://doi.org/10.5281/zenodo.17880363.
